# Mechanism of translation control of the alternative *Drosophila melanogaster* Voltage Dependent Anion-selective Channel 1 mRNAs

**DOI:** 10.1038/s41598-018-23730-7

**Published:** 2018-03-28

**Authors:** L. Leggio, F. Guarino, A. Magrì, R. Accardi-Gheit, S. Reina, V. Specchia, F. Damiano, M. F. Tomasello, M. Tommasino, A. Messina

**Affiliations:** 1 Department of Biological, University of Catania, Geological and Environmental Sciences, Catania, 95125 Italy; 2 Department of Biomedical and Biotechnological Sciences, University of Catania, Catania, 95123 Italy; 3 National Institute of Biostructures and Biosystems (INBB), Catania, Italy; 4 International Agency for Research on Cancer (IARC), World Health Organization, Lyon, 69372 France; 5 Department of Biological and Environmental Sciences and Technologies (DiSTeBA), University of Salento, Lecce, Italy; 6 IBB-CNR, Institute of Biostructure and Bioimaging, Section of Catania, Via Paolo Gaifami, 18-95126 Catania, Italy

## Abstract

The eukaryotic porin, also called the Voltage Dependent Anion-selective Channel (VDAC), is the main pore-forming protein of the outer mitochondrial membrane. In *Drosophila melanogaster*, a cluster of genes evolutionarily linked to VDAC is present on chromosome 2L. The main VDAC isoform, called VDAC1 (Porin1), is expressed from the first gene of the cluster. The *porin1* gene produces two splice variants, 1A-VDAC and 1B-VDAC, with the same coding sequence but different 5′ untranslated regions (UTRs). Here, we studied the influence of the two 5′ UTRs, 1A-5′ UTR and 1B-5′ UTR, on transcription and translation of VDAC1 mRNAs. In porin-less yeast cells, transformation with a construct carrying 1A-VDAC results in the expression of the corresponding protein and in complementation of a defective cell phenotype, whereas the 1B-VDAC sequence actively represses VDAC expression. Identical results were obtained using constructs containing the two 5′ UTRs upstream of the GFP reporter. A short region of 15 nucleotides in the 1B-5′ UTR should be able to pair with an exposed helix of 18S ribosomal RNA (rRNA), and this interaction could be involved in the translational repression. Our data suggest that contacts between the 5′ UTR and 18S rRNA sequences could modulate the translation of *Drosophila* 1B-VDAC mRNA. The evolutionary significance of this finding is discussed.

## Introduction

The voltage-dependent anion-selective channel (VDAC), also known as mitochondrial porin, is the most abundant protein found in the outer mitochondrial membrane of all eukaryotes. VDAC is the main gateway for the entry and exit of mitochondrial metabolites^1–3^, and thus it is suspected to control energetic exchanges between the mitochondria and the rest of the cell^4–6^. Porin/VDAC interacts with several kinases^7–9^ or structural proteins of the cytoskeleton, such as microtubule-associated protein 2^10^, tubulin^11^ and the dynein light chain Tctex-1^12^. Furthermore, porin/VDAC mediates the Ca^2+^ traffic in the mitochondria^13,14^. The role of VDAC in cancer is well known. VDAC1 contributes to the phenotype of cancer cells, regulating cellular energy production and metabolism^4,15,16^. Indeed, this protein is overexpressed in many cancer types, and silencing of VDAC1 expression induces an inhibition of tumour development. Among others, VDAC1 controls, together with other proteins, the release of the pro-apoptotic factors from mitochondria, e.g. cytochrome c^17–19^. VDAC1 can also regulate mitochondria-mediated apoptosis by interacting with hexokinases I and II and with proteins of the Bcl2 family, some of which are also highly expressed in many cancers^20,21^. Moreover, the involvement of VDAC1 in many neurodegenerative diseases, such as amyotrophic lateral sclerosis^22,23^, Parkinson’s^24^, Huntington’s^25^ and Alzheimer’s^26^, has also been widely proven.

Lower and higher eukaryotic cells express different sets of porin. In the budding yeast, *Saccharomyces cerevisiae*, two different porin genes have been identified, *POR1* and *POR2*^27^. In higher eukaryotes, like the mouse and the human, three porin/VDAC proteins are expressed^28–30^. The structures of mouse and human VDAC1^31,32^ and that of zebrafish VDAC2^33^ have been resolved and found to exhibit a folding pattern that is similar overall. In contrast, there is a general consensus that each of the VDAC isoforms has distinct physiological roles, because they could specifically interact with different proteins or be differentially sensitive to oxidation by reactive oxygen species^34^.

**Figure 1:**
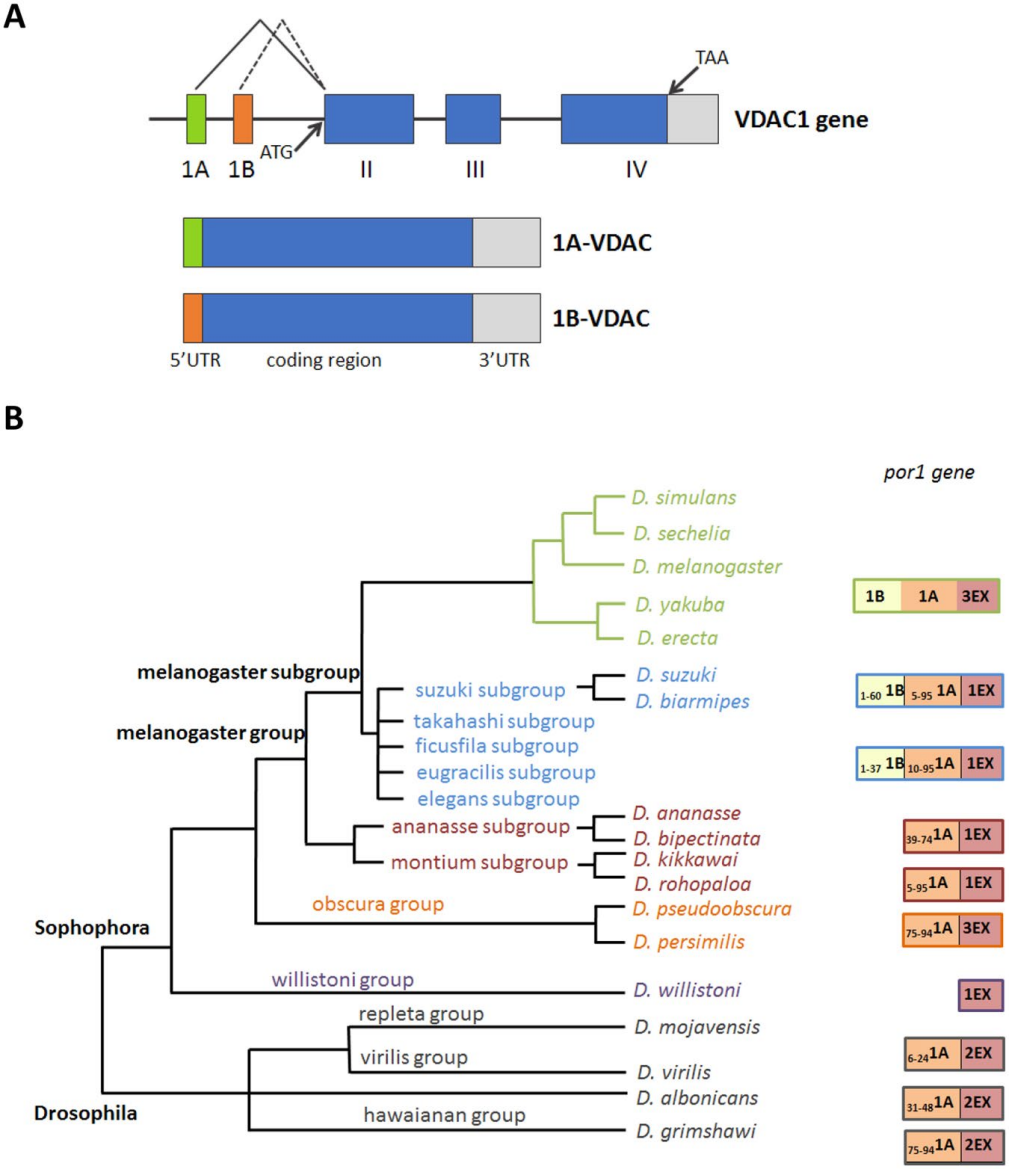
The *D. melanogaster* VDAC1 gene. (**A**) Schematic representation of the VDAC1 gene structure with its two alternative mRNAs. (**B**) Evolution promoted the introduction of the 1B-5′UTR into the VDAC1 gene, as can be seen by the progressive accumulation of its sequence within the more recent subgroup of the *Drosophila* genus.

In *Drosophila melanogaster*, the genomic locus 2L 32B 3–4 displays a cluster of four spatially close genes that are evolutionarily linked to VDAC^35^. These genes share the same exon-intron organization and are very likely to be the result of gene duplication events. However, the main known protein is the product of the first gene^36^. The second gene in the sequence, *porin2*, was shown to be expressed *in vivo* (by histological stainings)^37^ and to be able to form permeable pores (using the recombinant protein)^37^. The *porin1* gene, which produces VDAC1, is encoded by two main transcripts, 1A-VDAC1 and 1B-VDAC1. These transcripts show two alternative 5′ untranslated regions (UTRs) (corresponding to alternative exons 1A or 1B) but the same protein-encoding open reading frame (ORF) and the same 3′ UTR sequence ending at one of three different alternative polyadenylation sites (Fig. 1A). The ORF is for VDAC1, the pore-forming protein of the fly. Thus, in the fly, two alternative splice variants (transcripts) are expressed and are present at all fly development stages and in the same tissues, at the same time, but 1A-VDAC is more abundant^38^. VDAC1 from *D*. *melanogaster* has been purified, and its gene has been cloned, sequenced and mapped^36–39^. This protein shows very conserved functional features^36^, and it is indeed about 60% identical to the VDAC mammalian isoforms^38^.

The presence of alternative 5′ UTRs raised interest and was further investigated. *Drosophila* transposable P elements, when inserted into the *porin* locus, abolished VDAC expression and were found to produce a lethal phenotype^35^. Imprecise excision of such P elements showed that deletion of exon 1B and of its flanking sequences apparently has no effect on normal fly development or on VDAC protein level, whereas deletion of exon 1A suppresses VDAC protein expression^35^. It was therefore suggested that 1B-VDAC could be an unproductive transcript.

Here, we investigated the function of the two alternative *Drosophila* 5′ UTRs. Overall, our results suggest that a specific mechanism could be involved in the 1B-VDAC translational regulation. The biological significance of this mechanism is discussed.

## Results

### Conservation of 1A- and 1B- 5′ UTRs in the *Drosophila* genus

To evaluate the importance of the 1A- and 1B-5′ UTRs in the regulation of the *D*. *melanogaster porin1* gene expression, we performed a bioinformatic analysis to test the evolutionary conservation of these sequences in the *Drosophila* genus. The analysis performed in FlyBase revealed that the 1A- and 1B-5′ UTR sequences are statistically significantly present (E value < 0.05) in several species of the *Drosophila* genus (Fig. 1B).

The 1B-5′ UTR and the 1A-5′ UTR, or part of them, are simultaneously present in the same 11 subgroups (out of 18) belonging to the *Drosophila* genus, *Sophophora* subgenus. In particular, only in the *melanogaster* subgroup we detected the presence of both the whole 1B- and 1A-5′ UTR sequences, whereas in all the other *Sophophora* subgenus species, where the 1A-5′ UTR sequence was found to be partially present, the 1B-5′ UTR is completely or partially missing, containing only the starting sequence 1–37 or 1–60 (Fig. 1B). Overall, the evolutionary analysis indicates that the 1B-5′ UTR appeared only in the most recent species.

### Expression of *D*. *melanogaster* 1A-VDAC and 1B-VDAC cDNAs in VDAC-deficient yeast cells

**Figure 2:**
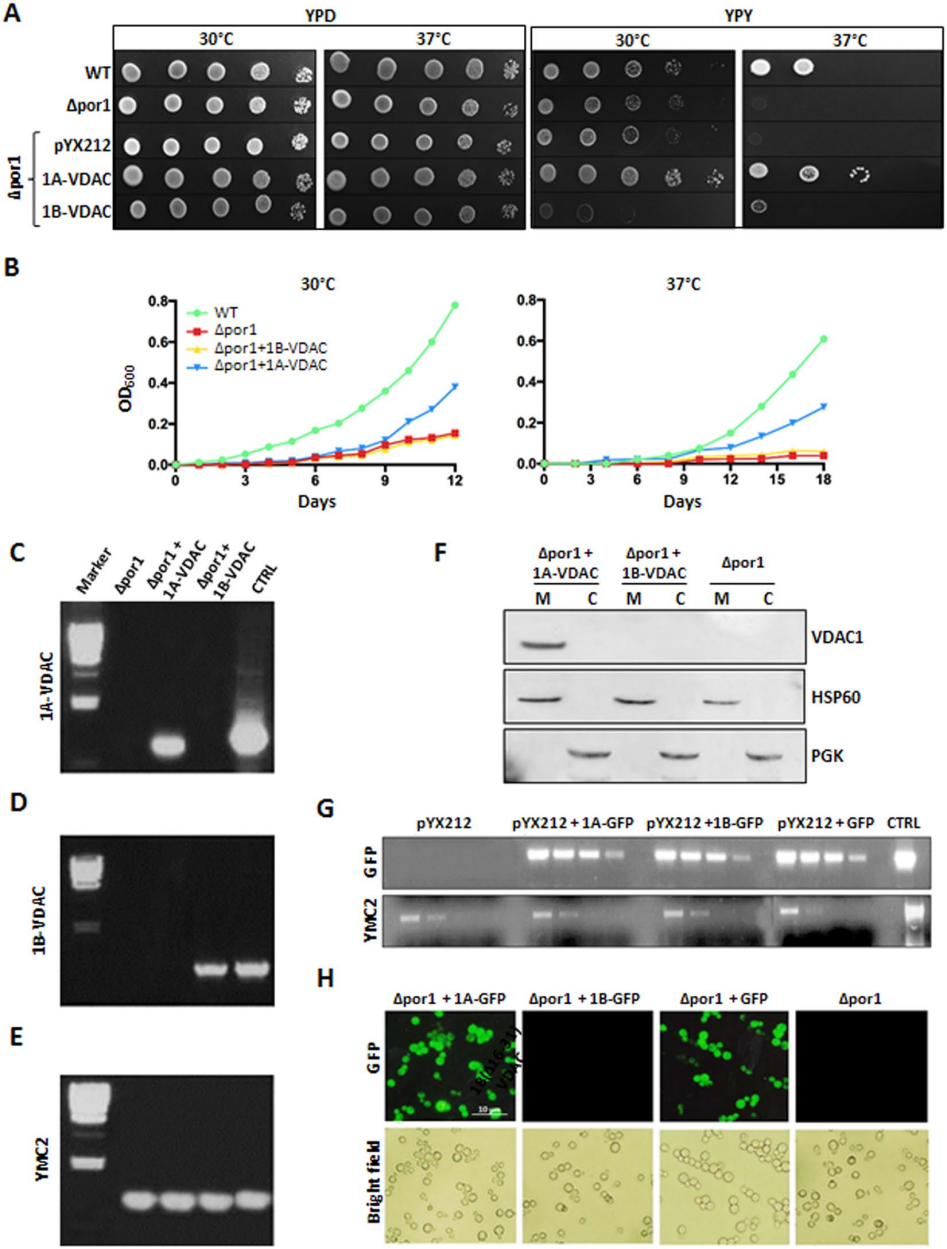
*D. melanogaster* 1A-VDAC and 1B-VDAC mRNAs are both transcribed in the VDAC-deleted Δpor1 yeast strain, but only 1A-VDAC is translated and its product targeted to mitochondria. (**A**) Representative drop-serial dilutions assay (*n* = 3) of *Δpor*1 yeast transformed with *D*. *melanogaster* 1A-VDAC or 1B-VDAC or empty vector pYX212; WT and *Δpor1* strains were used as controls. Yeast samples were plated on YPD or YPY and incubated at 30 °C or 37 °C from 2–6 days. No major differences were observed in yeast growth on YPD, at both temperatures. In contrast, a significant impairment of *Δpor*1 growth rate on YPY was observed when cells were transformed with 1B-VDAC or empty vector, but not with 1A-VDAC at not permissive temperature. (**B**) Representative yeast growth curves of *n* = 3 independent experiments of WT and *Δpor*1 yeast strain transformed as previously indicated. Cell growth was achieved on SG at 30 °C or 37 °C for 12 days and calculated by monitoring the optical density. Again, cell growth of *Δpor1* yeast transformed with 1A-VDAC was restored compared with control cells and with transformed 1B-VDAC *Δpor1* cells. Green, WT cells; red, *Δpor1* cells; blue, *Δpor*1 transformed with pYX212 carrying 1A-VDAC; yellow, *Δpor1* transformed with pYX212 carrying 1B-VDAC. (**C–E**) Transcription analysis in yeast of *D*. *melanogaster* 1A-VDAC and 1B-VDAC mRNAs. RNA extracted from *Δpor1* yeast transformed with 1A-VDAC or 1B-VDAC mRNA was reverse-transcribed and amplified by PCR by using specific primers for each 5′ UTR sequence (**C**,**D**) and the housekeeping control gene YMC2 (Yeast Mitochondrial Carrier YBR104W) (**E**). For each experiment, the pYX212 plasmid carrying the sequence 1A-VDAC (**C**), 1B-VDAC (**D**) and YMC2 (**E**) was used as positive control. The λ-HindIII molecular weight marker was used as reference. (**F**) Western blot analysis of mitochondrial and cytosolic lysates of *Δpor1* yeast strain expressing *D*. *melanogaster* 1A-VDAC, 1B-VDAC or untransfected. The antibody anti-*Dm*Porin1 (1:500) was used to identify VDAC1, and anti-Hsp60 (1:1000, Abcam) and anti-PGK (1:500, Novex) yeast antibodies were used respectively as mitochondrial and cytosolic controls. Only recombinant *D*. *melanogaster* 1A-VDAC. protein was expressed and directed to mitochondria of *Δpor1* yeast cells, but not *D*.*melanogaster* 1B-VDAC. (**G**) Reverse-transcription PCR of *D*. *melanogaster* 1A- and 1B-5′ UTR sequences fused to the coding sequence of GFP and cloned in pYX212 plasmid. *Δpor1* yeast cells were transformed with pYX212–1AGFP, pYX212–1BGFP or pYX212-GFP or pYX212 empty vector. Reverse-transcription PCR of GFP was performed using scalar dilution. Amplification of YMC2 was carried out from the same templates as loading control, and GFP amplification was used as a positive control. The mRNA GFP expression was detected under control of both 1A and 1B-5′ UTRs. (**H**) Fluorescence microscopy images of *Δpor1* yeast cells expressing GFP constructs. Yeast cells were grown in synthetic liquid media and observed by fluorescence or light microscopy. GFP fluorescence was detected exclusively in *Δpor1* cells carrying 1A-GFP-pYX212 but not in *Δpor1* yeast cells with 1B-GFP-pYX212.

*D*. *melanogaster* and other multicellular eukaryotes contain more active genes for VDAC isoforms. In these organisms, the alternative VDAC isoforms can be subject to an adaptive control of the total porin content^40^, whereas in yeast VDAC1 is the only functional porin expressed. Moreover, it is known that ΔVDAC flies are not viable^35^. On the basis of these assumptions, we preferred to investigate the function of the alternative 1A- and 1B-VDAC *D*. *melanogaster* transcripts first in a yeast strain lacking the yeast chromosomal VDAC gene, the *S*. *cerevisiae* strain (Δ*por1*, a kind gift of M. Forte, Oregon)^41^. Δ*por1* cells are unable to grow on a non-fermentable carbon source, such as glycerol, at 37 °C. The advantage in using this mutant yeast strain is that its growth defect can be complemented by the expression of a heterologous VDAC^28^. Transformation of Δ*por1* with *D*. *melanogaster* 1A-VDAC recovered the ability of yeast to grow on glycerol at 37 °C (Fig. 2A,B). In contrast, Δ*por1* cells transformed with 1B-VDAC were not able to grow under the same conditions (Fig. 2A,B).

We also determined the levels of the transcriptional and translational products of 1A-VDAC and 1B-VDAC in the Δ*por1* yeast strain (Fig. 2). Δpor1 cells transformed with 1A-VDAC or 1B-VDAC constructs were harvested, and total RNA was extracted and retro-transcribed by polymerase chain reaction (PCR) using primers designed on specific 1A- or 1B-5′ UTR sequences (Fig. 2C,D). Figure 2C,D confirm that both the 1A-VDAC and 1B-VDAC sequences were transcribed in yeast. PCR with primers amplifying a housekeeping gene was performed on the same templates, as a positive control (Fig. 2E).

The same cells were then analyzed for the presence of VDAC protein by means of a specific polyclonal antiserum. Figure 2F shows a western blot of proteins extracted from mitochondrial and cytosolic fractions of Δ*por1* cells transformed with 1A-VDAC or with 1B-VDAC. The immunostaining shows a clear reaction only for the mitochondrial fractions obtained from Δ*por1* transformed with 1A-VDAC. No protein band was detected in fractions from cells transformed with 1B-VDAC (Fig. 2F). These results indicate that 1A-VDAC mRNA is translated, and the protein is correctly targeted to mitochondria (Fig. 2F); accordingly, this transformed strain complements the phenotype associated with the deletion of the yeast porin gene (Fig. 2A). In conclusion, only 1A-VDAC mRNA produces a functional porin, whereas the 1B-VDAC mRNA is not translated.

### The *D*. *melanogaster* 1B-5′ UTR sequence is sufficient to inhibit the translation of a downward GFP coding sequence in yeast

To discriminate whether the inhibitory effect on the translation of the 1B-5′ UTR required the VDAC1 ORF, we fused the 1A- or 1B-exon upstream of the green fluorescent protein (GFP). These sequences were then cloned in pYX212 to obtain the reporter plasmids pYX212–1AGFP and pYX212–1BGFP. Δ*por1* yeast cells were transformed with the GFP pYX212 constructs, and the expression was analyzed by retro-trascription PCR (RT-PCR), fluorescence microscopy and western blot. A semi-quantitative PCR analysis showed that 1A-GFP and 1B-GFP transcripts were expressed at similar levels (Fig. 2G). Strikingly, no GFP fluorescence could be detected in cells transformed by pYX212–1BGFP (Fig. 2H). These results indicate that the presence of the 1B-5′ UTR has an inhibitory effect on the translation, independently of the nature of the downstream coding sequence.

### Does a uORF element located inside the 1B-5′ UTR sequence inhibit translation?

**Figure 3:**
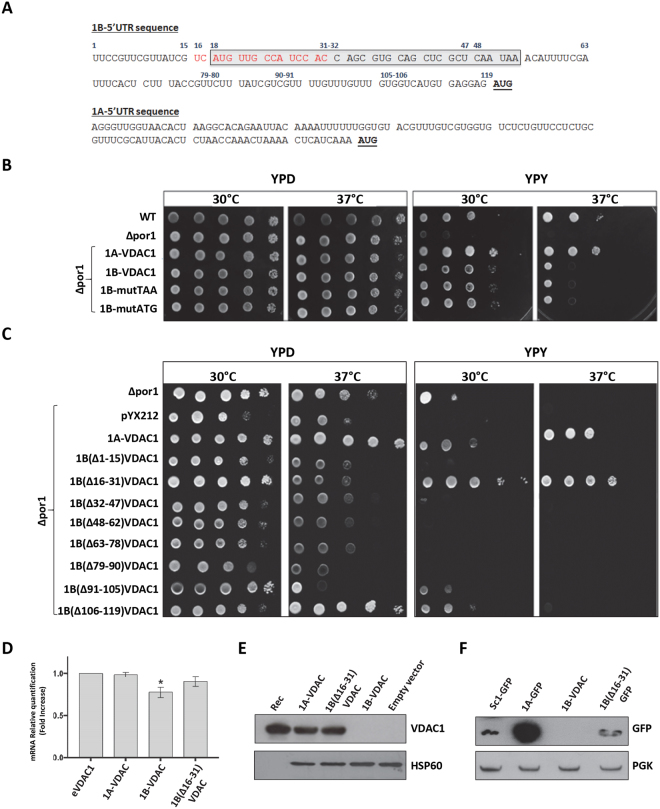
The translation of *D. melanogaster* VDAC protein is restored in Δpor1 yeast expressing the mutant 1B(Δ16–31)-VDAC. (**A**) Schematic representation of 5′ UTR sequences of 1A- and 1B-VDAC mRNAs. Grey, uORF sequence; red, 16–31 nucleotide control sequence; blue, specific nucleotide positions; underlined codons correspond to the first AUG of the VDAC coding sequence. (**B**) uORF in 1B-5′ UTR does not inhibit VDAC expression. Representative panel of a drop-serial dilutions assay of *Δpor1* transformed with 1B-VDAC or with 1BmutATG-VDAC or 1BmutTAA-VDAC. Yeast samples were plated on YPD or YPY and incubated at 30 °C or 37 °C for 2–6 days. No major differences were identified between yeast cells on YPD. The growth rate impairment typical of *Δpor1* in YPY was not rescued by transformation with 1BmutATG-VDAC or 1BmutTAA-VDAC, or with 1B-VDAC at 37 °C. (**C**) The 16-31 sequence in the 1B-5′ UTR is responsible for translational inhibition of 1B-VDAC. Representative panel of a drop-serial dilutions assay of *Δpor1* yeast transformed with 1B-VDAC deletion mutants. Each mutant was obtained by sequential deletion of 15 nucleotide stretches in the 1B-5′ UTR. Yeast samples were plated on YPD or YPY and incubated at 30 °C or 37 °C for 2–6 days. Cell viability of *Δpor1* on YPY was fully restored upon transformation with 1BΔ16–31-VDAC mutant, at both temperatures. (**D**) Quantitative analysis (qRT-PCR) of 1A-, 1B- and 1B(Δ16–31)-VDAC mRNAs in *Δpor1* cells. Endogenous VDAC1 (eVDAC), 1A-, 1B- and 1B(Δ16–31)-VDAC mRNA levels were normalized to the expression level of the housekeeping gene actin. A relative quantification of expression level was performed, using eVDAC as a control. A significant reduction in 1B-VDAC mRNA level was observed. Data were expressed as folding increase of the control (±SD). (**E**) Deletion of nucleotide stretch 16-31 in 1B-5′UTR restores translation of VDAC protein. Western blot analysis of mitochondrial fractions from *Δpor1* yeast lysate transfected with the same constructs as in (**D**). The antibody anti-*Dm*Porin1 (1:500) was used to identify VDAC1, and anti-Hsp60 (1:1000, Abcam) was used as mitochondrial control. RecVDAC1 is the purified recombinant DmVDAC, used here as a control. (**F**) Deletion of nucleotide stretch 16–31 in 1B-5′ UTR restores translation of GFP protein. Western blot analysis of Δ*por1* yeast lysates from transformed cells with 1A-GFP-pYX212, 1B-GFP-pYX212 or 1B-(∆16–31)-GFP-pYX212. Samples prepared as in (**D**) were tested for the GFP protein expression using a mouse anti-GFP antibody (1:1000, Roche).

Because the 1B-5′ UTR contains sufficient information to inhibit the translation of the downstream coding sequence, we searched for elements responsible for this effect. From a detailed analysis of the 1A- and 1B-5′ UTR sequences, we identified a single upstream ORF (uORF) of 12 codons in the 1B-5′ UTR, located 66 nucleotides before the canonical ATG (Fig. 3A).

The uORFs are prevalent cis-regulatory sequence elements in the transcript leader sequences of eukaryotic mRNAs^42^. From yeast to human, the uORFs mainly repress downstream translation by creating a functional barrier that cannot be easily overcome by pre-initiation complexes (PIC) that, upon their translation, undergo full ribosomal recycling, or by inhibiting progression of the ribosomes translating some of these uORFs^42,43^. Nevertheless, uORFs do not always repress translation of the main ORF, and the ribosome often, after uORF translation, can re-initiate downstream with a certain efficiency^42^. Therefore, the regulatory impact of a uORF on the translation of the downstream ORF has to be experimentally determined. To test the putative involvement of the 1B-5′ UTR uORF in translation, we mutagenised by the ‘‘QuikChange II XL Site-Directed Mutagenesis Kit” (QIAGEN) the start (AUG) or stop (UAA) codons of this uORF in the ACG and GCA codons, respectively. Thus, we looked for possible changes in the translational behaviour of the main ORF. The corresponding mutant constructs (named mutATG and mutTAA) were used to transfect the *∆porin* yeast strain and perform the complementation assay, under non-fermentative conditions (Fig. 3B). Figure 3B shows that suppression of the upstream ORF in the 1B-5′ UTR does not improve VDAC1, because the transformed yeast cells cannot complement the lack of the endogenous porin. These results confirm that the uORF element in 1B-5′ UTR does not contribute to the translation control mechanism of 1B-VDAC mRNA.

### A specific sequence in the 1B-5′ UTR element controls the translation

To identify one or more putative control elements located in the 1B-5′ UTR, we performed a mutagenesis scanning experiment producing eight 1B-5′ UTR mutants, each with the deletion of a continuous sequence of 15 nucleotides with no overlap, thus covering the whole 1B-5′ UTR (Fig. S1). Next, each mutant was cloned in pYX212 and used for the *Δpor1* yeast complementation assay (Fig. 3C). In this assay, Δ*por1* yeast transfected with 1A-VDAC was used as a positive control, and Δ*por1* yeast, not transformed at all or transformed with pYX212 empty vector, was used as a negative control. As expected, all 1B-5′ UTR mutants displayed major problems to grow on glycerol at 30 °C, like the non-transfected Δ*por1* strain (Fig. 3C). This behaviour became more evident when cells were grown on glycerol at 37 °C, under restrictive conditions (Fig. 3C). Interestingly, only the 1B(Δ16–31)-VDAC mutant was able to fully complement the growth defect of Δ*por1* yeast, as well as Δ*por1* yeast transformed with 1A-VDAC (positive control) (Fig. 3C). All the other 1B-mutants had the same behaviour as the *Δpor*1 yeast; also, 1B(Δ91–105)- and 1B(Δ106–119)-VDAC showed feeble growth at 30 °C, but they are unable to recover the growth defect of the porin-lacking mutant at 37 °C, and therefore they do not produce a VDAC protein. The Δ*por1* yeast expressing 1B(Δ16–31)-VDAC is apparently able to even better complement the mutant phenotype compared with 1A-VDAC (Fig. 3C). The reason for this is unknown.

Therefore, we paid specific attention to the analysis of the Δ*por1* yeast carrying the 1B(Δ16–31)-VDAC mutant. In particular, we analyzed the cells for the presence of transcription and translation products from the 1B(Δ16–31)-VDAC construct. VDAC transcripts were present at similar levels in Δ*por1* yeast transformed with 1B- or 1B(Δ16–31)-VDAC constructs, as found by quantitative real-time PCR (qRT-PCR) assay (Fig. 3D). The expression of VDAC protein was tested showing a strong immune reaction in extracts from the Δ*por1* strain carrying 1B(Δ16-31)-VDAC or 1A-VDAC, but not in the same cells transformed with the 1B-VDAC sequence (Fig. 3E). Using GFP as a reporter, we found that also Δ*por1* transformed with 1B(Δ16-31)-GFP recovered the GFP expression (Fig. 3F), confirming the relevance of this sequence. Our results allow us to suppose that the 1A-5′ UTR sequence contains elements that increase the mRNA translation in yeast. Indeed, the protein expression in Fig. 3F was highly increased when the GFP coding sequence was linked to 1A-5′ UTR, compared with a construct containing only the GFP coding sequence.

We also produced 1B(Δ19–31)-, 1B(Δ19–28)- and 1B(Δ16–28)-VDAC deletion mutants, to test whether a subset of 16-31 nucleotides from the 1B-5′ UTR sequence is preferentially responsible for the translation control or rather the whole sequence was necessary. Results from this experiment (Fig. S2) show that the removal of 16-28, or 19–28 or 19–31 nucleotides from the 1B-5′ UTR sequence just barely began to restore the inhibitory effect of VDAC synthesis. Surprisingly, even though partial mutants lack only few nucleotides of the 16–31 region, the deletion of the whole 16–31 region is necessary to fully recover the translation of the main ORF in the 1B-VDAC mRNA.

### 1B-5′ UTR mRNA contains motifs putatively recognised by different RNA-binding proteins

The RBPMap server (http://rbpmap.technion.ac.il/) was used to identify the presence of RNA motif(s) able to bind known RNA-binding proteins (RBPs)^44^. Using as a query the 1A- or 1B-5′ UTR sequences, under default search conditions (see Methods), we found many putative RBPs shared by 1A- and 1B-5′ UTRs and few RBPs specific for each target RNA (Table S2). Interestingly, about 70% of the putative RBPs recognised both 1A and 1B sequences, but no RBP specific for 1B-5′ UTR specifically recognised the 16–31 sequence.

Next, we performed a RNA electrophoretic mobility shift assay (REMSA) with the aim of detecting yeast RBPs able to bind an RNA oligo (called the 10–37 oligo) from 1B-5′ UTR. The 10–37 RNA oligo corresponds to the 10–37 sequence of the 1B-5′ UTR and thus contains the 16–31 sequence that we suspected to be involved in the 1B-VDAC mRNA translational control (see the Section above). Total extracts from wild-type M3 cells were incubated with the 10–37 oligo, and with an RNA negative control oligo, corresponding to the 74–95 sequence of the 1B-5′ UTR (the 74–95 oligo). Figure S3A,B shows that yeast proteins specifically interact with the 10–37 RNA oligo. In particular, this target produced two bands with delayed mobility, probably resulting from its interaction with two different RBPs or, alternatively, with more molecules of the same RBP on different motifs in the 10–37 RNA target.

REMSA analysis was also performed on *Drosophila* SL2 cell extracts using the same RNA oligos (Fig. S3C,D). Specific interactions were revealed between the 10–37 RNA and *D*. *melanogaster* protein extracts, producing a pattern consistent with the distribution of RNA bands produced with yeast extracts (Fig. S3C). The outcome of this experiment underlines that in *Drosophila* there are probabily more proteins able to specifically interact with the 10–37 sequence of the 1B-5′ UTR than in yeast.

Futhermore, to isolate the ribonucleoprotein complexes produced by the interaction of specific yeast proteins with the 10–37 RNA oligo, we performed RNA pull-down experiments (see Methods). Proteins interacting with RNA oligos were then resolved by SDS-PAGE. It must be taken into account that the yield of purified protein was very low. Therefore, after Coomassie gel staining, each protein band was excised from the gel lane and processed (Fig. S4). We cut out 9 bands from the eluate of 10–37 RNA and, in parallel, 9 bands from the control (74–95 RNA) (Fig. S4). Gel bands were trimmed and trypsinised, and then analysed by mass spectrometry (MS) for protein identification. The list of proteins with a high score and high coverage identified by MS analysis is reported in Table S3. There was no conventional yeast RBP but, interestingly, we found some proteins that are engaged in the control of translation, such as Asc1 and eIF4A.

### The 16–31 sequence of the 1B-5′ UTR influences the translation in *Drosophila*

**Figure 4:**
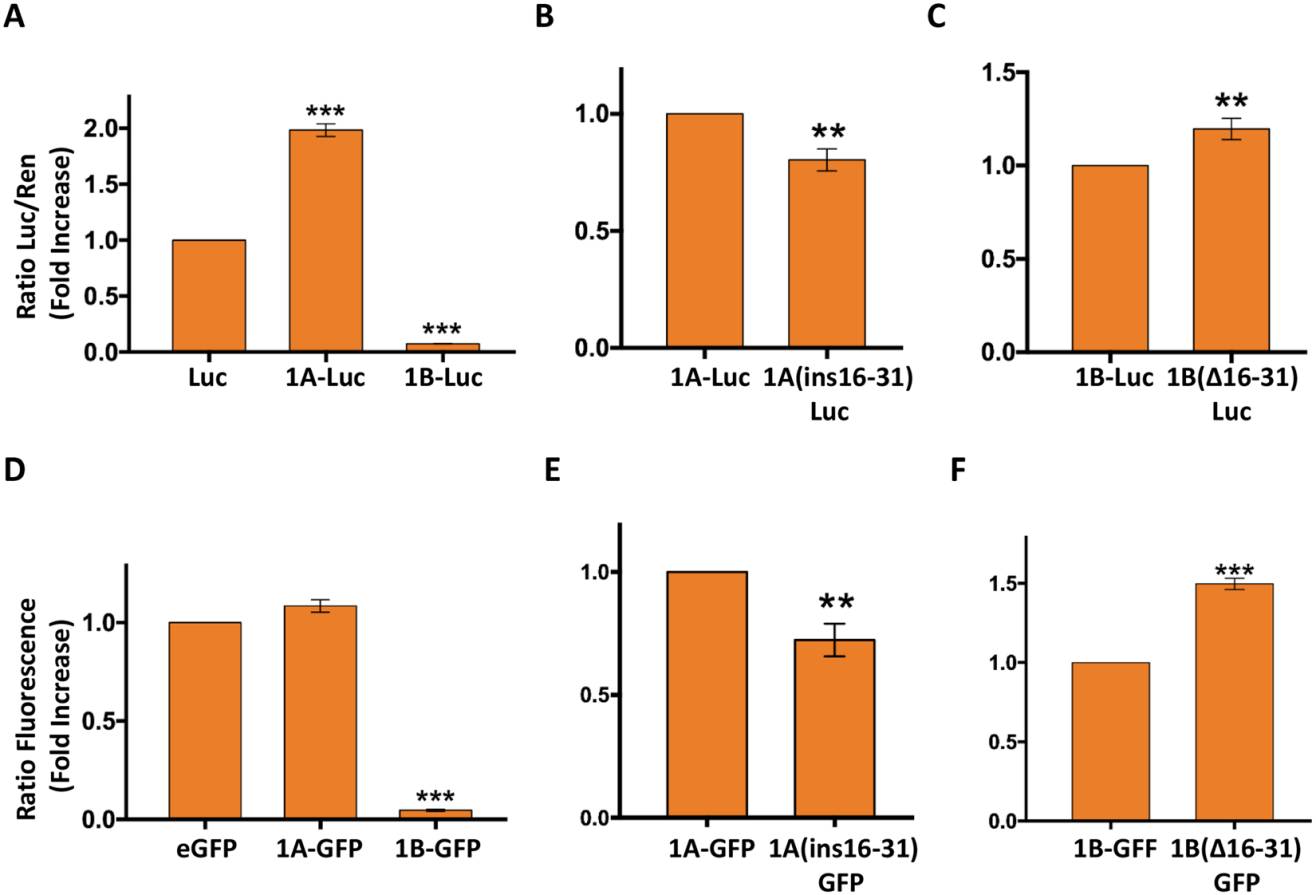
Expression of transcripts carrying mutant or wild type 1A- or 1B-5′ UTR in *D. melanogaster* and mammalian cells. (**A–C**) Analysis by luciferase assay of *D*. *melanogaster* SL2 cells transfected with constructs carrying alternative 1A- or 1B-5′ UTR sequences. Cells were transfected with pMK26 vector carrying WT luciferase (Luc), 1A-Luc, 1B-Luc, 1A(ins16–31)-Luc or 1B(Δ16–31)-Luc. 1A(ins16–31) indicates a mutated 1A-5′ UTR where the 16-31 sequence of 1B-5′ UTR was inserted. Cells were co-transfected with *Renilla* luciferase (Ren). Data are expressed as fold increase of Luc/Ren ratio and as means ± SD (*n* = 3). (**A**) Relative quantification of 1A-Luc and 1B-Luc as fold increase of Luc (control). Whereas the presence of the 1 A sequence significantly enhances the Luc/Ren ratio, the presence of 1B results in a dramatic reduction in the expression. ***p < 0,001 compared with Luc. (**B**) Relative quantification of 1A(ins16–31)-Luc compared with 1A-Luc (control). A small but significant reduction was observed in the 1A(ins16–31)-Luc sample. **p < 0,01 compared with 1A-Luc. (**C**) Relative quantification of 1B(Δ16–31)-Luc compared with 1B-Luc (control). A significant increase was observed in 1B(Δ16–31)-Luc. **p < 0,01 compared with 1B-Luc. (**D–F**) Analysis of GFP expression in HeLa cells transfected with constructs carrying alternative 1A- or 1B-5′ UTR sequences. GFP expression analysis in HeLa cells transfected with constructs containing the 1A- or 1B-5′UTR sequences. Exactly, 1A, 1B, 1A(ins16–31) or 1B(Δ16–31) sequences were cloned in frame with GFP. These constructs were used for cell transfection. Data are expressed as fold increase of the relative fluorescence and as mean ± SD (*n* = 3). (**D**) Relative quantification of 1A-GFP and 1B-GFP as fold increase of GFP (control). The 1B sequence was able to dramatically reduce the GFP signal. ***p < 0,001 compared with GFP. (**E**) Relative quantification of 1A(ins16–31)-GFP in comparison to 1A-GFP (control). A small but significant reduction was observed in the 1A(ins16–31)-GFP sample. **p < 0,01 compared with 1A-GFP. (**F**) Relative quantification of 1B(Δ16–31)-GFP compared with 1B-GFP (control). A significant increase was observed in 1B(Δ16-31)-GFP. ***p < 0,001 compared with 1B-GFP.

We evaluated in *D*. *melanogaster* cells the effect of the 16–31 sequence of 1B-5′ UTR on the translation of luciferase reporter gene. Embryonic SL2 cells were transfected with constructs expressing the luciferase sequence fused downstream of 1A-, 1B-, 1B(Δ16-31)- or 1A(*ins*16–31)-5′ UTR (Fig. 4A–C). In the latter construct, the 16–31 sequence of the 1B-5′ UTR was incorporated into the 1A-5′ UTR, at the same distance from the start codon as in the original sequence (1B-VDAC), substituting for the 1A-5′ UTR nucleotides. The luciferase assay on SL2 cell extracts showed that, in *Drosophila* as in yeast, the 1A-5′ UTR and 1B-5′ UTR sequences increase and extinguish the translation, respectively (Fig. 4A). Our data also confirmed the involvement of the 16–31 sequence of the 1B-5′ UTR in the translation control mechanism. Indeed, when the 16–31 sequence was inserted into the 1A-5′ UTR it resulted in a clear reduction of the reporter gene expression, about 25% less than for the construct expressing 1A-Luciferase (Fig. 4B). In contrast, when the 16–31 sequence was removed from the 1B-5′ UTR (Fig. 4C), the transcript recovered nearly 20% of translation, compared with the 1B-Luciferase transcript, which was totally untranslated (Fig. 4C). Similar results were also obtained in HeLa cells expressing constructs where GFP as a reporter gene was fused to the tested 5′ UTR sequences (Fig. 4D–F). Taken together, these results enable us to hypothesise that the control mechanism acting on the translation of the alternative 1B-VDAC mRNA in *D*. *melanogaster* is based not only on the action of the 16–31 sequence but probabily also on engagement of more factors. In comparison, the yeast machinery is probably less complex.

### In *Drosophila* SL2 cells, expression of 1A-VDAC and 1B-VDAC mRNAs is coordinated

**Figure 5:**
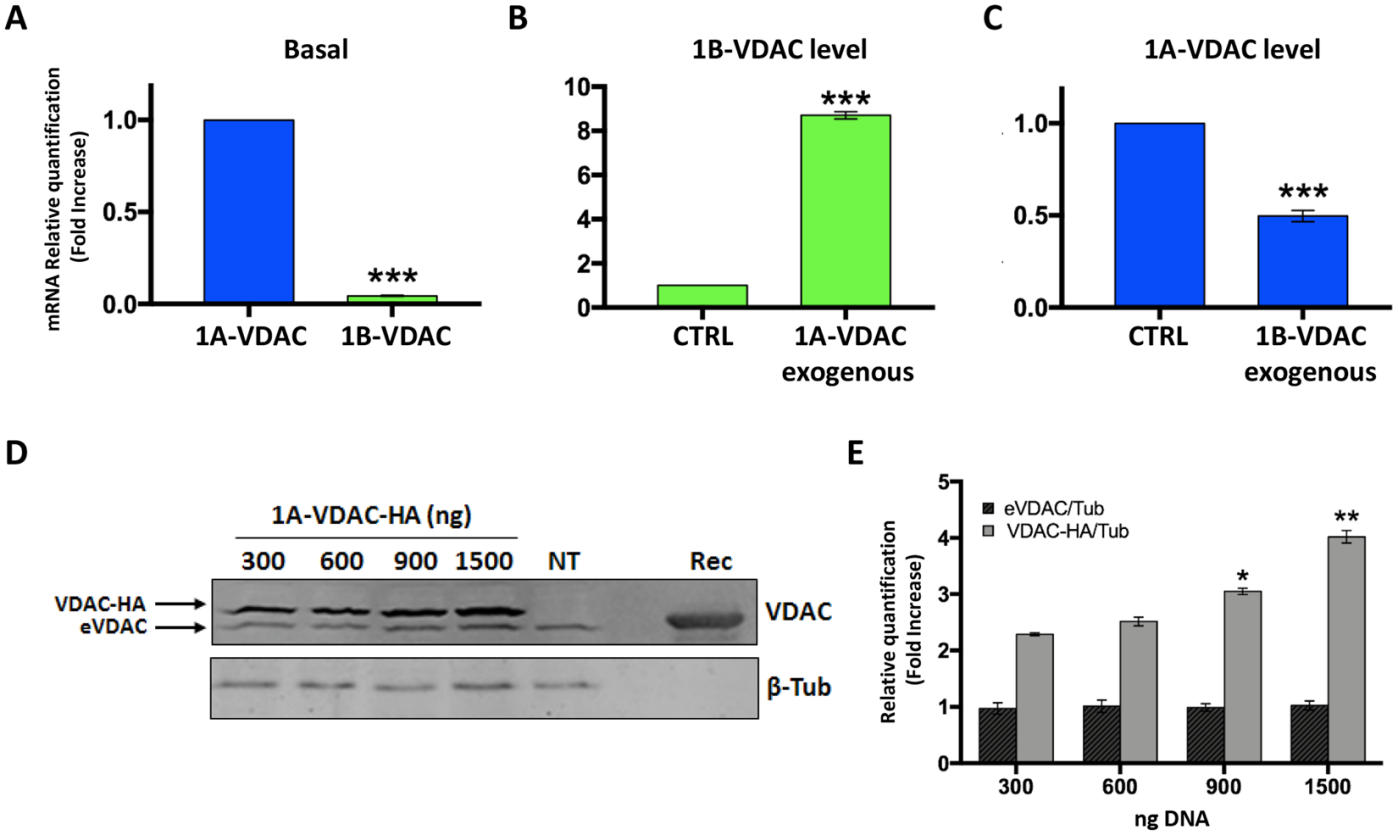
Expression levels of 1A-VDAC and 1B-VDAC are correlated. (**A–C**) qRT-PCR of VDAC mRNAs in *Drosophila* SL2 cells. Relative quantification of expression level of 1B-VDAC and 1A-VDAC mRNAs was performed after different stimuli. *Act1* was used as the housekeeping gene for normalisation of data. Data are expressed as folding increase of mRNA level and as mean ± SD (*n* = 3). (**A**) Relative amount of 1B-VDAC mRNA compared with 1A-VDAC, under basal conditions. 1B-VDAC mRNA amount was significantly lower than the 1A-VDAC mRNA amount. ***to p < 0,001 compared with 1A-VDAC. (**B**) The 1B-VDAC mRNA level observed after 1A-VDAC overexpression. As reported, a boost in 1A-VDAC mRNA level produces a significant increase in 1B-VDAC mRNA. ***to p < 0,001 compared with 1B-VDAC. (**C**) The 1A-VDAC transcript level detected after 1B-VDAC overexpression. The increase in 1B-VDAC mRNA significantly reduced the amount of 1A-VDAC transcript. ***to p < 0,001 compared with 1B-VDAC. (**D**) Western blot analysis of SL2 cell lysates transfected with increasing concentration of 1A-VDAC-HA. The anti-*Dm*Porin1 antibody (1:500) was used to identify both endogenous VDAC1 (eVDAC) and heterologous VDAC-HA, and the anti-Tub (1:500, Abcam) antibody was used as loading control. Rec referred to the purified recombinant DmVDAC, used here as a control. (**E**) Relative quantification of VDAC amount by densitometry. eVDAC) and VDAC-HA were normalised to the corresponding β-Tub signal. Data are shown as relative fold increase of eVDAC in untransfected cells (NT). Whereas eVDAC remained similar in all tested conditions, the amount of VDAC-HA increased depending on the DNA concentration used for transfection. *p < 0,05 and **p < 0,01 compared with VDAC-HA level at 300 ng DNA.

Under physiological conditions, in *D*. *melanogaster* embryonic SL2 cells, the 1B-VDAC transcript level is about 1/10th of 1A-VDAC (Fig. 5A). Our results also show that after overexpression of 1B-VDAC, the 1A-VDAC mRNA level decreases by about 50%, compared with the original amount. Similarly, the overexpression of the 1A-VDAC transcript produces a significant increase (about 80%) in the 1B-VDAC mRNA level. However, after overexpression of the 1A-VDAC transcript, the endogenous 1A-VDAC protein level does not change (Fig. 5D,E). This result thus underlines that 1B-VDAC mRNA is probably not involved in the control mechanisms of VDAC cellular concentration.

### The 5′ UTR 1B-VDAC mRNA interacts with 18S rRNA

To gain more insights into the mechanism of translation control by the 1B-VDAC 5′ UTR, we performed a bioinformatic analysis. Preliminarily, RNA sequences for 1A-VDAC (FlyBase ID FBtr0332029) and 1B-VDAC (FlyBase ID FBtr0080183) were submitted to the *mfold* web server (at http://unafold.rna.albany.edu) for prediction of RNA secondary structure. A similar pattern of secondary structures with comparable hybridisation energies was obtained for both *D*. *melanogaster* VDAC mRNAs.

**Figure 6:**
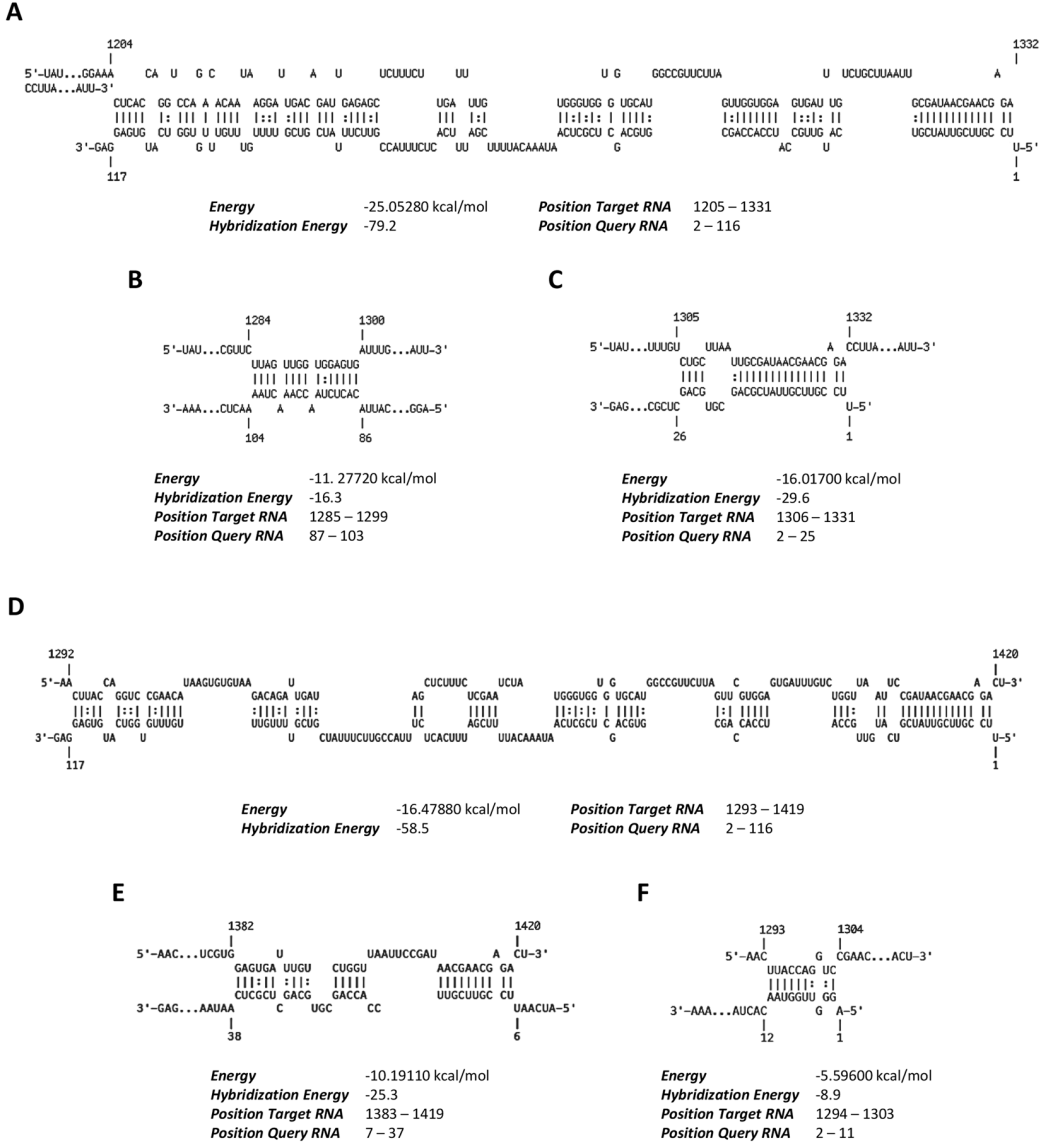
Predicted interactions between VDAC 5′ UTRs and 18S rRNA. (**A**) Comparison of predicted interactions between 1B-VDAC mRNA and yeast 18 S rRNA. The 2–116 region of 1B-5′ UTR is engaged in a high energy bound (−79.2 kcal/mol) with the 1205–1331 region of 18 S rRNA. (**B**) As in (**A**), interaction of 1A-VDAC mRNA with yeast 18S rRNA. The 1A-5′ UTR interacts with a short region of 18S rRNA with a weak hybridisation energy (−16.3 kcal/mol) and involving the 87–103 and 1285–1299 regions of 1A-VDAC and 18S rRNA, respectively. (**C**) As in (**A**), interaction of 1B(∆16–31)-VDAC mRNA with yeast 18S rRNA. When the 16–31 region of the 1B-5′ UTR is removed, the most significant interactions with the 18S rRNA are confined to a region of 20 bp in length from 2 to 25 in the 1B(∆16–31)-5′ UTR, showing a hybridisation energy of only −29.6 kcal/mol. (**D**) Comparison of predicted interactions between 1B-VDAC mRNA and *D*. *melanogaster* 18S rRNA. The 2–116 region from 1B-5′ UTR bounds with a high hybridisation energy (−58.5 kcal/mol) to the 1293–1419 nucleotide region of 18S rRNAd. (**E**) As in (**D**), interaction of 1A-VDAC mRNA interaction with *D*. *melanogaster* 18S rRNA. The 1A-5′ UTR is engaged in a low energy bound (−25.3 kcal/mol) with a short sequence of 18S rRNA. (**F**) As in (**D**), interaction of 1B(∆16–31)-VDAC mRNA interaction with *D*. *melanogaster* 18S rRNA. The mutant 1B(Δ16–31)-VDAC mRNA pairs with *D*. *melanogaster* 18S rRNA yielding a short dsRNA stretch with a smaller hybridisation energy (−8.9 kcal/mol) than 1B-VDAC mRNA. All the analyses were performed using IntaRNA software (http://rna.informatik.uni-freiburg.de/IntaRNA/Input.jsp).

Furthermore, IntaRNA software (http://rna.informatik.uni-freiburg.de/IntaRNA/Input.jsp) was used to predict the interactions between yeast 18S ribosomal RNA (18S rRNAy) and 1A-VDAC or 1B-VDAC mRNA. The 1B-(Δ16–31) mutant, studied in this work, was also examined. This analysis predicted that the 2–116 sequence of 1B-VDAC mRNA targets the 1205–1331 sequence of 18S rRNAy, producing a long double-stranded RNA (dsRNA) structure with a hybridisation energy of −79,2 kcal/mol (Fig. 6A). In contrast, the 87–103 sequence of 1A-VDAC mRNA is only able to pair with the 1285–1300 sequence of 18S rRNAy, leading to a very short dsRNA, with the low hybridisation energy of −16,3 kcal/mol (Fig. 6B). Similarly, 1B(Δ16–31)-VDAC mRNA can pair with 18S rRNAy at the 1306–1331 sequence, producing only a short dsRNA structure with a low hybridisation energy value (Fig. 6C).

IntaRNA software was also used to predict interactions between *D*. *melanogaster* 18S ribosomal RNA (18S rRNAd) and 1A-, 1B- or 1B(Δ16–31)-5′ UTRs. This additional analysis showed that the 2–116 sequence of 1B-VDAC mRNA interacts with the 1293–1419 region of 18S rRNAd, producing a dsRNA duplex with a hybridisation energy of −58.5 kcal/mol (Fig. 6D). This interaction area corresponds to the region rRNAy:1B-VDAC. The 1294–1303 sequence of 18S rRNAd, similar to 18S rRNAy, was able to pair with the very short 2–11 sequence of 1A-VDAC mRNA, with the low hybridisation energy of −8.9 kcal/mol (Fig. 6E). Also, the mutant 1B(Δ16–31)-VDAC mRNA pairs with 18S rRNAd yielding a short dsRNA duplex, again with a low hybridisation energy (Fig. 6F).

**Figure 7:**
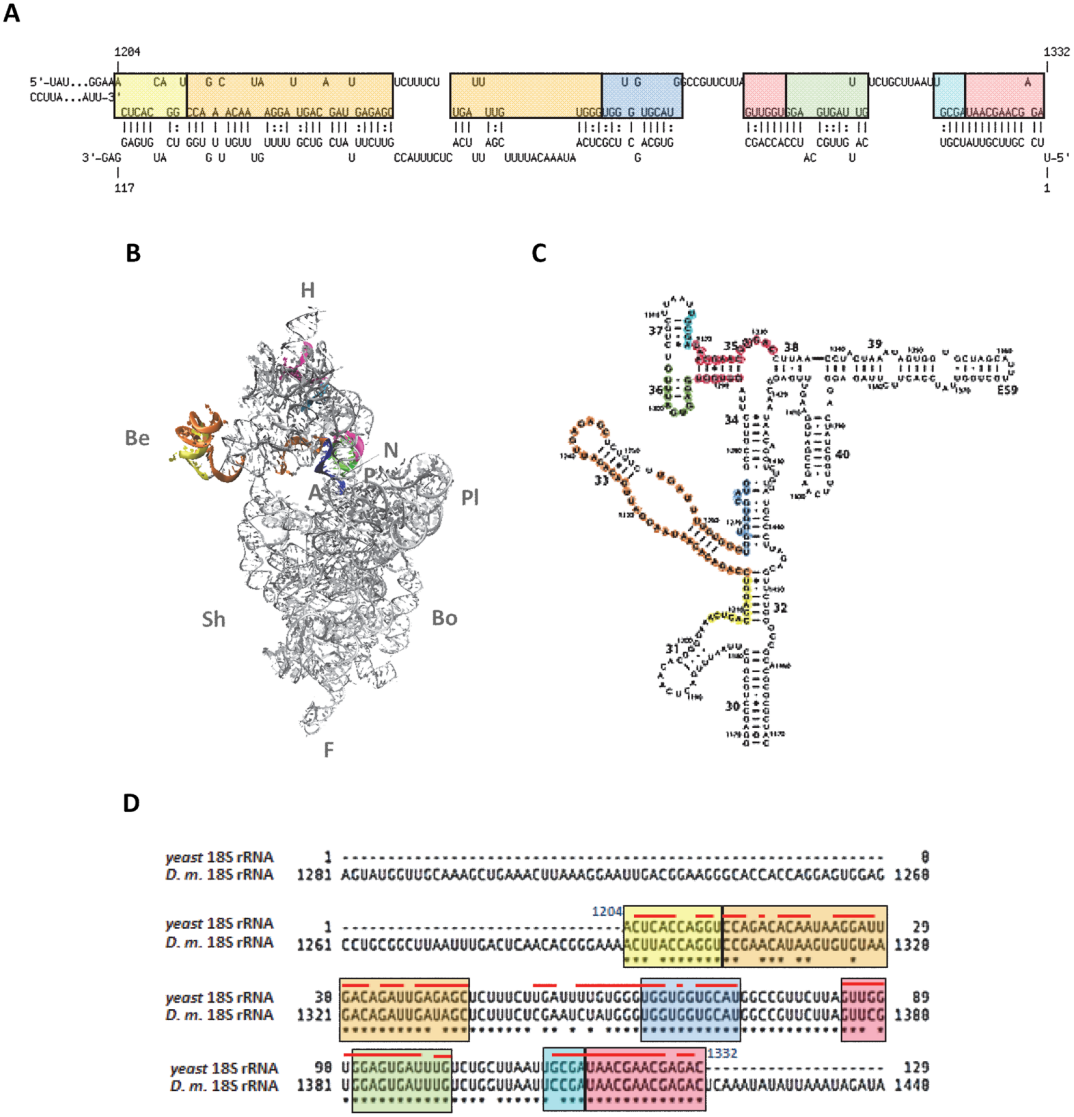
Interaction of 5′ UTR of D. melanogaster 1B-VDAC mRNA with specific regions of 18S rRNA. (**A**) Schematic of interactions between 1B-5′ UTR and yeast 18S rRNA obtained using intaRNA software (from Fig. 6A). Yellow, the 1204–1214 sequence; orange, the 1215–1246 and 1255–1266 sequences; blue, the 1267–1278 sequence; magenta, the 1288–1293 and 1330–1342 sequences; green, the 1294–1304 sequence; cyan, the 1325–1319 sequence. (**B**) Cartoon depicting the 18S rRNA tertiary structure at interface-side of the yeast 40S subunit (PDB file 3U5B was used for the representation). 18S rRNA specific sequences involved in the interaction with 1B-5′ UTR are coloured as in (**A**). Abbreviations: H, head; Be, beak; Pl, platform; Sh, shoulder; Bo, body; F, foot; A, site A; P, site P. (**C**) Secondary structure of a portion of the 3′ major domain of *S*. *cerevisiae* 18S rRNA showing the same sequence and colours as in (**A**). (**D**) Alignment of 18S rRNA sequences from *Saccharomyces cerevisiae* and *Drosophila melanogaster* putatively involved in the interaction with the 5′ UTR of 1B-VDAC mRNA.

Interestingly, the region of 1B-VDAC mRNA, predicted to interact with 18S rRNA of yeast or *D*. *melanogaster*, corresponds to the whole 1B-5′ UTR sequence (Fig. 6A,D). In particular, the helices 32–36 and part of helices 31 and 37 of 18S rRNA represent domains putatively involved in these interactions (Fig. 7A–C). These rRNA regions contribute to form the ribosomal 3′ major domain, located at the head of the 40S subunit, and these sequences are highly conserved in eukaryotes (Fig. 7B). Moreover, the rRNA sequences forming duplexes with the 5′ UTR of 1B-VDAC mRNA, represent 40S domains mainly exposed to solvent^45–47^, with helices 31–35 usually unbound to r-proteins^46^. In addition, it is highly significant that the helices 32 and 34 of 16S rRNA are known to protrude into the mRNA channel of the minor ribosomal subunit^47^, with the loop of helix 31 located under the 40SP site^45^. Therefore, the 5′ UTR from the alternative 1B-VDAC mRNA, after recognition from the minor ribosomal subunit, could easily establish interactions with many 18S rRNA domains, not bound to r-proteins and located in the molecular environment surrounding the mRNA channel of the 40S ribosomal subunit (Fig. 7B).

### Either 1A-VDAC and 1B-VDAC transcripts bind to *D*. *melanogaster* ribosomes, but only 1A-VDAC is actively translated

**Figure 8:**
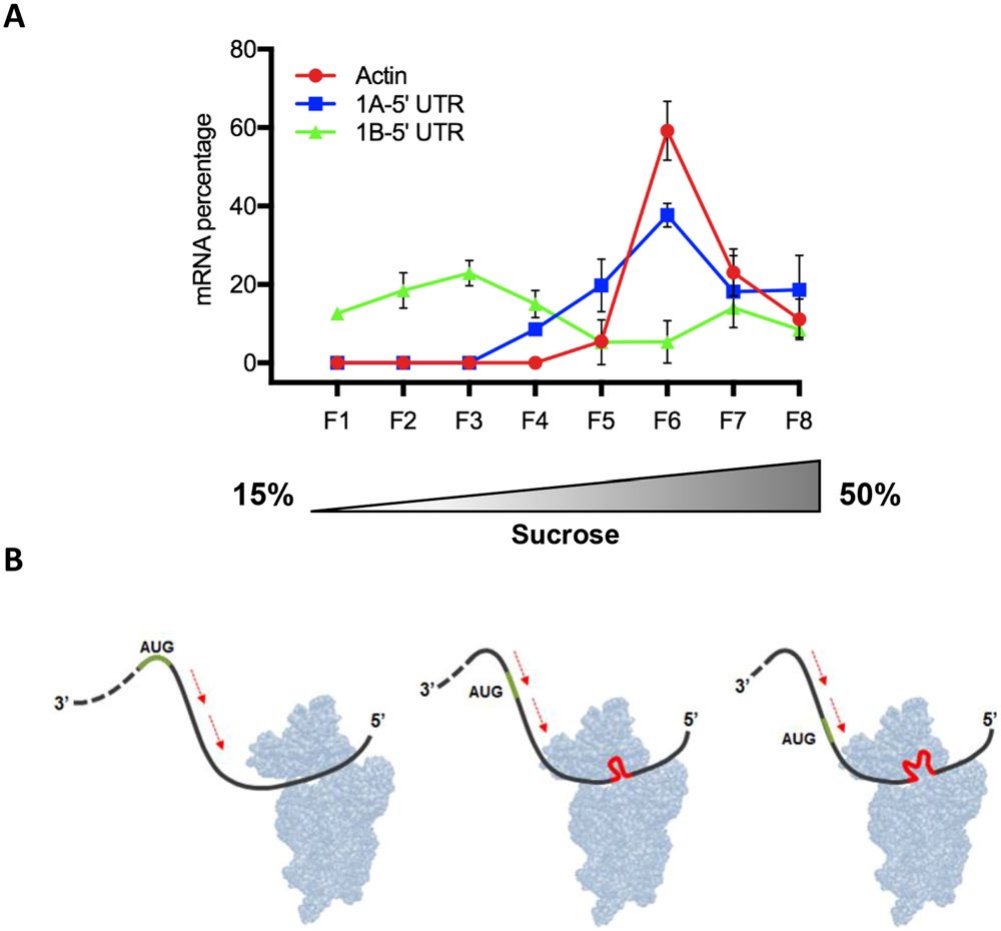
In Drosophila, 1A-VDAC mRNA is actively translated and 1B-VDAC mRNA is mainly untranslated. (**A**) Quantitative analysis of 1A-VDAC and 1B-VDAC mRNAs from polysome gradient of *Drosophila* embryos. mRNAs encoding for 1A-VDAC, 1B-VDAC and actin were detected by qRT-PCR, performed with specific primers (listed in Table S1), in each fraction of *D*. *melanogaster* polysome gradient. The distribution of each mRNA is shown along the gradient as a percentage of total mRNA and each value is expressed as mean ± SD of two independent polysome purification experiments. The mRNA encoding for 1A-VDAC is almost entirely associated with actively translating fractions (fractions 5–7, spanning real polysomes), whereas the 1B-VDAC transcript is mostly in the untranslated fractions (fractions 1–4, spanning mainly single ribosomal subunits), corresponding to untranslated mRNA. (**B**) A model of translation inhibition of the *D*. *melanogaster* 1B-VDAC mRNA depicting the delayed scanning of the 40S ribosomal subunit. As reported in the text, the model was mainly based on the strong interactions between the 1B-5′ UTR region and some exposed domains of 18S rRNA.

We used polysome profiling from *D*. *melanogaster* embryos to analyse the translational status of the two alternative VDAC mRNAs by quantitative RT-PCR analysis on individual fractions of the polysome gradient. The results show that mRNAs coding for 1A-VDAC were present in fractions at the bottom of the gradient, suggesting that these mRNAs are associated with polysomes and thus are actively translated (Fig. 8A). In contrast, 1B-VDAC mRNAs were detected especially in the first fractions of the polysome gradient, suggesting that they are mainly in a translationally repressed state, with only few molecules of 1B-VDAC mRNA recruited onto polysomes (Fig. 8A).

Figure 8B shows a model taking into account the results of the influence of 1B-VDAC mRNA on the ribosome translation initiation presented herein.

## Discussion

In this work, we focused on the regulation of expression of VDAC1 in *D*. *melanogaster*. In this species, the *porin1* gene produces two alternative transcripts named 1A-VDAC and 1B-VDAC, containing an identical coding sequence but two completely different 5′ UTRs. To gain further insights into the biological function of these two alternative splicing forms of VDAC, we introduced them into a VDAC-lacking system, an established *S*. *cerevisiae* strain where the *porin1* gene was inactivated (∆*por1* strain). The advantage of the yeast cell is its viability (under fermentative conditions), whereas *D*. *melanogaster* cells cannot survive the deletion of the VDAC1 gene.

In ∆*por1* yeast, the heterologous 1A-5′ UTR directed transcription and translation of VDAC and of GFP used as a reporter; in contrast, the 1B-5′ UTR directed the transcription but not the translation of the VDAC or the reporter gene. These results confirm that only the 1A-VDAC, but not the 1B-VDAC, is able to complement the growth defect of the ∆*por1* yeast cells. We obtained similar data also in *Drosophila* cells by using a luciferase reporter gene downstream of the 1A- or 1B-5′ UTR. Our results suggest that the 1B-5′ UTR affects VDAC expression by inhibiting protein translation. Furthermore, our results suggest that this mechanism is independent of the coding region cloned downstream of the 5′-UTR.

### Understanding the mechanism

We aimed to understand the mechanism responsible for the negative influence of the 1B-5′ UTR on the translation of the coding sequences fused downstream. Gene expression in eukaryotic cells is regulated at multiple levels, including mRNA translation. Such control allows rapid changes in protein concentrations and, thus, it is used to maintain cellular homeostasis. Most translation regulation is exerted at the very first stage, when the AUG start codon is identified after the 5′ UTR ribosome scanning. Consequently, any occurrence that prevents or inhibits the ability of the ribosome to scan the 5′ UTR reduces the efficiency of translation initiation. Many mechanisms able to produce this effect are well known^48,49^. Therefore, we assayed some of them, such as the presence of uORFs or stable secondary structures and the association with regulatory RBPs.

We ruled out the possibility that the small uORF located in the 1B sequence is involved in translational control. Our bioinformatic analysis suggested that no putative strong secondary structure in the untranslated region of 1B-VDAC mRNA should be involved in the inhibition of translation. In addition, our bioinformatic predictive analysis of RBPs showed that there is no known RBP specific for the 1B-5′ UTR, although we are aware of the limitations of computational tools. Moreover, we did not consider the possible involvement of miRNAs, because the 3′ UTR of 1B-VDAC is included in the corresponding 3′ UTR of 1A-VDAC, which is longer. Therefore, because a regulatory mechanism involving a miRNA action targeted to this region of 1B-VDAC mRNA could not be specific for the 1B-mRNA, we ruled it out.

### Involvement of translational apparatus

Using a mutagenesis scanning approach, we identified the 16–31 nucleotide region of the 1B-5′ UTR sequence as responsible in yeast for the inhibitory effect on translation. The defect in the growth of the ∆*por*1 yeast strain was indeed complemented when the strain was transformed with 1B(∆16–31)-VDAC mutant, underlining that its removal is sufficient to re-establish the translation. We also verified that the 16–31 sequence works similarly in *Drosophila*, although the translation inhibition must rely also on others factors. Therefore, by MS analysis of the proteins bound to an RNA oligo containing the 10–34 sequence of the 1B-5′ UTR, we searched for proteins directly or indirectly involved in the translation control. In particular, we recognised eIF4A, eIF5a and Asc1 (Table S3). eIF4A is a RNA helicase working in the first stage of translation as a subunit of the cap-binding complex eIF4F, which unwinds the RNA secondary structures in the 5′ UTR^50^. Asc1/RACK1 associates with the 40S subunit close to the mRNA exit channel, where it interacts with eIF4E of eIF4F^51^. Asc1/RACK1 is involved in the control of the translation of housekeeping genes^52^ and, in general, represses gene expression^53^. It is known that RACK1 loss-of-function mutations cause early developmental lethality in the mouse and the fly^54,55^, like VDAC knockout organisms. Moreover, in yeast, loss of ASC1 reduces translation of mitochondrial r-proteins and, like for lack of VDAC1, causes cells to be unable to use non-fermentable carbon sources, demonstrating a direct control of ASC1 on mitochondria functionality^52^. Interestingly, RACK1 has many interaction partners, ranging from kinases and signalling proteins to membrane-bound receptors and ion channels. Thus, under stress conditions^56^, RACK1 can function as a signalling hub of newly synthesised proteins.

From this viewpoint, we can hypothesise that in yeast the 16–31 sequence might prevents eIF4A function, maybe trapping eIF4A in an inactive conformation. In *Drosophila*, 1B-VDAC translation could be repressed at the starting point by the coordinated action of more molecules, probably recruited *in situ* by RACK1. We also identified Gus1, which together with Arc1, is known to form a protein complex operating in the control of translation^57^. In addition, the presence of two different heat-shock proteins (Hsp12 and Hsp76) in this pool of interacting proteins should indicate their recruitment after stress conditions.

### Contacts between ribosome and 5′ UTRs

We also tested the ability of the 1A- and 1B-5′ UTR sequences to contact protein-free domains of 18S rRNA, the only rRNA in the 40S subunit. Because 18S rRNA mutations impair the integrity of the scanning-competent pre-initiation complex and/or its joining together with the 60S subunit^45^, the translation initiation rate might be reduced by strong and long-range interactions between the protein-free domains of 18S rRNA and the 5′ UTR(s) of the incoming mRNA. It has already been demonstrated in eukaryotes that gene expression regulation at the level of translation may occur thanks to specific interactions between mRNAs and rRNA domains. In particular, a highly specific sequence complementarity between 18S rRNA and the 5′ UTRs of mRNAs across species has been predicted^58^; this complementarity may modulate the scanning processivity of the 40S subunit through the 5′ UTR of mRNAs, which could even stall the initiating PICs in the case of long-range interactions.

In particular, by prediction analysis of RNA:RNA interactions between yeast 18S rRNA and the two alternative *D*. *melanogaster* VDAC mRNAs (1A-VDAC and 1B-VDAC), we found that, in yeast as in *D*. *melanogaster*, almost the whole 1B-5′UTR sequence is able to strongly interact with a long sequence of 18S rRNA. In contrast, the 1B(∆16–31)-5′UTR sequence can only weakly interact with a short sequence of rRNA in the 40S subunit, thus showing a behaviour similar to that 1A-5′ UTR. These results underline the relevance of 1B-5′ UTR and, in particular in yeast, of its 16–31 sequence for the mechanism of translation control. Interestingly, we also found that some regions of the rRNA sequence involved in the interaction with the 1B-5′ UTR fold in solvent-exposed domains, and some of them are turned towards the mRNA path of the ribosome 40S subunit (Fig. 7B)^47^. Therefore, these rRNA domains should be able to contact the 5′ UTR in the incoming 1B-VDAC mRNA, producing a stop in the ribosome scanning. It is noteworthy that a sequence of about 35 nucleotides can be allocated inside the ribosomal mRNA path of PIC and that we found that almost the whole 1B-5′ UTR sequence, (2–116 nucleotides), may potentially interact with three 18S rRNA helices (helix 35, helix 36 and a portion of the helix 34) arranged near the mRNA path at the neck of 40S (Fig. 7B,C). In addition, the large helix 33, together with parts of helix 31 and helix 32, being arranged at the beck of the 40S subunit, could easily interact with the 1B-5′ UTR. In this way, the 1B-VDAC mRNA translation rate would be negatively controlled by its 5′ UTR sequence through the collective action of several interactions with 18S rRNA, the result of which would be a strong delay in ribosome scanning of 1B-VDAC (for a model of this mechanism, see Fig. 8B). Probably, this effect in *Drosophila* could also be the result of additional interactions with fly-specific proteins, ribosomal or not. In any case, it is extremely relevant that the sequences encompassing these rRNA helices are highly conserved between *S*. *cerevisiae* and *D*. *melanogaster;* this indicates that the mechanism we have described in a mixed yeast-fly system is likely to act in *D*. *melanogaster*.

### What is the physiological role of 1B-VDAC mRNA?

VDAC is an essential but dangerous protein. Its function as a pro-apoptotic factor is well known^4^ and therefore it is essential for the cell to implement a suitable control of VDAC protein level. Also, specific conditions of cell growth involving high energy demand are known to induce up-regulation of VDAC^59^ associated with the requirement of mitochondrial biogenesis. Furthermore, these events must be coordinated with the expression of the other mitochondrial proteins, codified by the nuclear genome and from mitochondrial DNA. Therefore, it is conceivable to suppose the presence in the cell of a “sentry” molecule able to sense, directly or indirectly, the amount of this crucial protein. We demonstrated that in *Drosophila* the level of 1B-VDAC transcript is highly increased as a result of overexpression of 1A-VDAC mRNA. When the level of the 1B-VDAC transcript was increased by its overexpression, the endogenous 1A-VDAC mRNA level was meaningfully reduced. Importantly, our results show that the unproductive 1B-VDAC mRNA is able to respond to 1A-VDAC transcript levels, and thus it might work as a molecule signalling the need for activation of mitochondrial biogenesis. This hypothetical role of 1B-VDAC mRNA is supported by its interaction with Asc1/RACK1. Asc1/RACK1 responds to multiple signals, and might act to coordinate the expression of other mitochondrial proteins and thus affect cell respiration.

In addition, the assignment of this important role to 1B-VDAC mRNA might help us to understand why the evolution of the *Drosophila* genus proceeded towards the acquisition of an alternative 5′ UTR with specific features.

In conclusion, our results extend our earlier reports^35,36,38,39^ and provide further evidence that in *D*. *melanogaster* the 1A-VDAC transcript is responsible for protein expression, while the alternative 1B-VDAC mRNA is not active in this respect. Moreover, in this work we show that a specific mechanism could be responsible for the translation inhibition of the alternative *D*. *melanogaster* 1B-VDAC1 transcript.

## Methods

### Mitochondria preparation and RNA isolation from yeast cells

A litre culture of *S*. *cerevisiae* was grown for 2 days in YP medium supplemented with 2% galactose and 0,1% glucose. Cells were washed twice with sterilised water and resuspended in buffer 1 (100 mM Tris/H_2_SO_4_ pH 9.4 + 10 mM DTT) at 2 ml/g of cells. The suspension was incubated at 30 °C for 20 minutes- under slow shaking. Cells were harvested by centrifugation and subsequently resuspended in 1.2 M sorbitol and centrifuged at 3000 × *g* for 5 minutes at 25 °C. To obtain sphaeroplasts, cells were resuspended in buffer 2 (20 mM KH_2_PO_4_ pH 7.4 + 1.2 M sorbitol) at 7 ml/g of cells and 1 mg/g of cells. Zymolyase 100 T (Amsbio) was added. The mixture was incubated for 1 hour at 30 °C and shaken into a glass flask at 70 rpm. To eliminate debris, sphaeroplasts were centrifuged and washed twice with 1.2 M sorbitol. The pellet was weighted and resuspended (6.5 ml/gr of pellet) in ice-cold buffer 3 (0.6 M sorbitol + 10 mM Tris/HCl pH 7.4 + 1 mM PMSF + 1 mM EDTA pH 7.4) and homogenised. Mitochondria were isolated by three consecutive centrifugations for 5 minutes at 1500 × *g* at 4 °C, 5 minutes at 3000 × *g* at 4 °C and 15 minutes at 12000 × *g*. The resulting pellet was resuspended in buffer 4 (0.25 M sucrose + 20 mM tris/HCl pH 7.4 + 1 mM EDTA pH 7.4) and homogenised again. Then, 30 ml of buffer 4 was added to the homogenate and centrifuged for 5 minutes at 3000 × *g*. The supernatant was re-centrifuged for 20 minutes at 12000 × *g* and the mitochondrial pellet was resuspended in 0.5–1 ml of buffer 4.

Total RNA was isolated from yeast cells and then reverse transcribed as described previously^60^. The cDNAs were used for real-time PCR experiments.

### RNA assays

#### RNA electrophoretic mobility shift assay (REMSA)

A 3′-biotin labelled RNA oligonucleotide corresponding to 10–37 sequence in the 1B-5′UTR (Biotin-10–37 RNA, sequence 5′-UAUCGUCAUGUUGCCAUCCACCAGCGU-3′), was synthesised by a company (Biolegio BV). A RNA oligonucleotide with the same sequence but unlabelled (10–37 RNA) was also synthesised as a competitor. In addition, RNA oligos biotinylated and non-biotinylated at 74–95 in the 1B-5′UTR sequence were obtained. Total proteins were purified from the M3 yeast strain. REMSA analysis was performed using the “LightShift Chemiluminescent RNA EMSA (REMSA) Kit” (Thermo Scientific) according to the manufacturer’s instructions, with slight modifications. The RNA-protein interactions were improved by adding 31 µg of heparin to each sample. The biotin-labelled 10–37 RNA was used at 0.5 nM, and 6 µM of unlabelled 10–37 RNA was used to test the specificity of the RNA-protein interaction. Four micrograms of yeast proteins were used for any assay. The final concentration was 2 nM for the biotin-labelled 74–95 RNA and 6 µM for the unlabelled 74–95 RNA.

#### RNA pull down assay

Dynabeads MyOne Streptavidin C1 beads (Invitrogen) were used to bind the biotin-labelled RNA to the streptavidin of the beads. Then, 400 pmol of biotin-labelled 10–37 or 74–95 RNA was bound to 200 µg of magnetic beads in the presence of binding and washing buffer 2X (10 mM Tris/HCl pH 7.5, 1 mM EDTA and 2 M NaCl). After 1 hour under slow shaking, the samples were washed three times with binding and washing buffer 1X and suspended in the same buffer. In another tube, 160 µg of M3 yeast proteins, prepared as previously described, was mixed with 1.5X REMSA buffer (100 mM HEPES pH 7.3, 200 mM KCl, 10 mM MgCl_2_ and 10 mM DTT), 2 µl of 100 mM DTT, 3 µl of tRNA (10 µg/µl) in DEPC water and 100 µg of pre-washed beads. After 1 hour under slow shaking, the unbound proteins were transferred into the tube containing the RNA:beads complexes and the mixture was slowly shaken for 1 hour. Subsequently, ultraviolet (UV) cross-linking was performed by placing the tube underneath the light bulb of a 254 nm UV light source. The RNA-protein complexes were irradiated for 10 minutes, the supernatant was collected and the beads were resuspended in 20 µl of 1X LDS and boiled for 5 minutes. Samples were electrophoresed in a 12% polyacrylamide SDS-PAGE.

#### Quantitative real-time PCR (qRT-PCR)

RNA was isolated from cells using the “ReliaPrep RNA Cell Miniprep system” (Promega). Then, 2 µg of RNA was retrotranscribed using the “QuantiTect Reverse Transcription kit” (QIAGEN). Real-time PCR experiments were performed in triplicate using the “QuantiFast SYBR Green PCR kmeit” (QIAGEN). Amplification was performed using the following thermocycling program: 95 °C for 5 minutes, 40 cycles of denaturation at 95 °C for 15 seconds, 60 °C for 20 seconds, 68 °C for 30 seconds. Specific primers were designed to amplify 1A-VDAC, 1B-VDAC and actin transcripts (Table S1). Values from qRT-PCR were expressed as mean ± SEM, representatives of three sets of independent experiments. Data were statistically analysed by one-way ANOVA or t-test.

### Polysome fractionation and RNAs purification

About 1000 *Drosophila* wild-type embryos were dechorionated and incubated with 0.1 mg/ml cycloheximide in PBS 1X for 10 minutes on ice. The embryos were homogenised with a motorised plastic pestle in lysis buffer (20 mM Tris/HCl pH 7.4, 140 mM KCl, 5 mM MgCl_2_, 0.5 mM DTT, 1% Triton X-100, 0.1 mg/ml cycloheximide, 50 U/ml RNasin) and incubated for 10 minutes on ice. After centrifugation at 12000 × *g* for 10 minutes at 4 °C, the supernatants (about 300 µg of RNA) were gently layered on the top of a 15–50% w/w sucrose gradient. A sucrose density gradient of 12 ml (made in 20 mM Tris/HCl pH 7.4, 5 mM MgCl_2_, 140 mM KCl, 0.5 mM DTT, 100 µg/ml cycloheximide, 20 U/ml RNasin,) was prepared in polyallomer tube and centrifuged at 35000 rpm for 160 minutes, at 4 °C, in a Beckmann Coulter Optima L-90K ultracentrifuge using a SW-41 rotor^61^. For the quantification of transcripts from the polysome gradient, the sucrose gradient was divided into 8 fractions of 1,5 ml each from the top of the gradients. The RNA was recovered from each fraction using Trizol reagent (Invitrogen), according to the standard protocol, and was then precipitated with one volume of isopropanol. The RNA, after centrifugation at 13000 rpm for 30 minutes, was washed with 70% ethanol and then was resuspended in RNase-free water. An aliquot of each fraction was used to check RNA quality on agarose gel. Finally, purified RNA was used for cDNA synthesis by using SuperScript III (Life Technologies) according to the standard protocol. qRT-PCR analysis was performed as previously described.

### Cell culture and transfection

*Drosophila melanogaster* embryonic SL2 cells were cultured in Schneider’s *Drosophila* medium + L-glutamine (Gibco) supplemented with 10% FBS (Gibco) and 1% penicillin-streptomycin (Gibco), and incubated at 25 °C without CO_2_.

For RNA isolation from untransfected or transfected SL2 cells, 3 × 10^6^ cells per well were seeded into 6-well plates. After 24 hours, cells were transfected with 600 ng of 1A-VDAC or 1B-VDAC in pAc5.1 vector by using Lipofectamine 3000 (Invitrogen) according to the manufacturer’s instructions. After incubation for 48 hours, cells were processed for RNA extraction.

For western blotting analysis, 3 × 10^6^ SL2 cells were seeded. After 24 hours, cells were transfected with different concentrations of 1A-VDAC in pAc5.1 vector, by using Lipofectamine 3000 (Invitrogen). After 48 hours of incubation, cells were processed for protein extraction; 100 µg of each total lysate was electrophoresed, blotted and immune-decorated with a specific polyclonal anti-*D*.*melanogaster* porin antiserum (1:500). The anti-tubulin antibody (1:500, Sigma Aldrich) was used as control.

For the luciferase assay, 3 × 10^6^ SL2 cells were seeded on a 6-well plate. After 24 hours, cells were co-transfected with 600 ng of constructs expressing VDAC sequences fused to luciferase, and 20 ng of *Renilla* Luciferase plasmid, by using Lipofectamine 3000 (Invitrogen). After incubation for 48 hours, cells were processed for the luciferase assay using the “Dual Glo Luciferase Assay System” (Promega) according to the manufacturer’s instructions. Values from the luciferase assay were expressed as mean ± SEM, representatives of three sets of independent experiments performed in triplicate. Data were statistically analysed by one-way ANOVA.

For GFP expression analysis from human cells transfected with VDAC constructs, HeLa cells were cultured in DMEM (Gibco) supplemented with 10% FBS (Gibco) and 1% penicillin/streptomycin (Gibco), at 37 °C in an atmosphere of 5% CO_2_ in air. HeLa cells were seeded on a 12-well plate and transfected after 24 hours by using 1 µg of DNA and Lipofectamine 3000 (Invitrogen). Cells were analysed 24 hours after transfection by flow cytometry.

### Bioinformatic analysis

The pairings between 5′ UTRs and the yeast or *Drosophila* 18 S rRNA were generated with the “IntaRNA” tool (http://rna.informatik.uni-freiburg.de/IntaRNA/Input.jsp), setting up standard conditions.

### Statistical analysis

Significance was determined as reported and indicated as *p < 0.05, **p < 0.01, and ***p < 0.001.

All primers used in this work are listed in Table S1 (Supplementary Informations).

## Electronic supplementary material

Supplementary materials
