# Supplementary material for: Mechanism of translation control of the alternative Drosophila melanogaster Voltage Dependent Anion-selective Channel 1 mRNAs
Source: Sci Rep. 2018 Mar 28;8:5347. doi: 10.1038/s41598-018-23730-7 (PMC5871876; doi:10.1038/s41598-018-23730-7)
Supplement: Supplementary file 1 — Supplementary materials [file 41598_2018_23730_MOESM1_ESM.pdf]

Mechanism of translation control of the alternative *Drosophila melanogaster* Voltage Dependent Anion Channel 1 mRNAs.

L. Leggio[1,2+], F. Guarino[2,3+], A. Magrì[1], R. Accardi-Gheit [4], S. Reina[1,2], V. Specchia[5], F. Damiano[5], M.F. Tomasello[6], M. Tommasino[4], A. Messina[1,3*]

[1] University of Catania, Department of Biological, Geological and Environmental Sciences, Catania 95125, Italy.

[2] University of Catania, Department of Biomedical and Biotechnological Sciences, Catania 95123 Italy.

[3] National Institute of Biostructures and Biosystems (INBB), Catania Italy.

[4] International Agency for Research on Cancer (IARC), World Health Organization, Lyon 69372, France

[5] Department of Biological and Environmental Science and Technology (DiSTeBA) - University of Salento - Lecce, Italy.

[6] IBB-CNR, Institute of Biostructure and Bioimaging, Section of Catania, Via Paolo Gaifami, 18 - 95126, Catania, Italy.

[+] These authors contributed equally to this work.

*Correspondence to: Angela Messina, Department of Biological, Geological and Environmental Sciences, Section of Biochemistry and Molecular Biology, University of Catania, Italy; email: mess@unict.it

Supplementary Information

Supplementary Methods

Cloning of recombinant cDNAs and constructs

VDAC constructs. The alternative 1A-VDAC and 1B-VDAC cDNAs used in this work have been previously isolated in our lab (ref. 35 in the text). The 1A-VDAC cDNA was subcloned in the pYX212 plasmid, a shuttle vector containing a 2 µ plasmid replication origin, the triose phosphate isomerase promoter and the URA 3-selectable marker. The 1B-VDAC cDNA was subcloned in pYX212 vector. Mutants of 1B-VDAC cDNA were obtained using the "QuikChange II XL Site-Directed Mutagenesis Kit" (QIAGEN) on a wild-type 1B-VDAC sequence subcloned in pBS-K (Stratagene). All 1B-VDAC mutants were next subcloned in pYX212. The accuracy of the constructs was confirmed by DNA sequencing.

The *D. melanogaster* VDAC1 cDNA was cloned in pQE30 (Qiagen) and a tag of six histidines (His-tag) was fused to its N-terminal[39]. This construct was used to isolate recombinant Dm-VDAC1.

GFP constructs. GFP gene was cloned in pYX212 obtaining thus pYX212GFP construct. 1A- and 1B-UTR sequences were introduced at EcoRI/EcoRI sites, upstream of the GFP in pYX212GFP. The correct orientation and sequence of each UTR was confirmed by sequencing.

Cloning and expression in yeast of D.m. VDAC cDNAs. Alternative cDNAs, 1A-VDAC and 1B-VDAC, and mutants of 1B-VDAC cDNA, cloned in the pYX212, were intoduced into *Δpor1* by lithium-acetate transformation; the same strain was transformed with the corresponding empty vector as a control. The recombinant *D.m.* VDAC protein, used as control in western blotting experiments, was produced as in[1]. Expression of VDAC was evaluated in yeast cells by western blotting using a specific polyclonal anti-*Dm*Porin1 antiserum (1:500). The antibodies anti-HSP60 (1:1000, Abcam) and anti-PGK (1:500, Novex) were used as controls.

Cloning and expression in yeast of GFP-VDAC constructs. 1A-5'UTR, 1B-5'UTR and mutant 1B(Δ16-31)-5'UTR were cloned upstream of GFP in pYX212GFP and then were intoduced into wild type M3 strain by lithium-acetate transformation. Expression of GFP was evaluated in the total lysate of transformant yeast by western blotting using a mouse anti-GFP (1:1000, Roche).

Cloning and expression in Drosophila SL2 cells of VDAC constructs fused or not to luciferase reporter gene. 1A-VDAC and 1B-VDAC cDNAs were cloned EcoRI/HindIII in pAc5.1/V5-His (Thermo Fisher Scientific), a vector designed for transient expression of recombinant proteins from the constitutive promoter of the Drosophila actin 5C gene. The accuracy of the constructs was confirmed by DNA sequencing. The pMK26-Luciferase construct was obtained by cloning the firefly Luciferase gene into HindIII/XhoI sites of pMK26/ACTSV40BS, a vector for protein expression from the Drosophila actin 5C promoter. 1A-, 1B-, 1B(Δ16-31)-, and 1A(ins16-31)-UTRs were amplified by PCR using primers listed in Table S1 and then were cloned at EcoRV/HindIII sites of pMK26-Luciferase vector, upstream

luciferase gene. The mutant 1A(ins16-31)-VDAC was obtained using the "QuikChange II XL Site-Directed Mutagenesis Kit" (QIAGEN) on the pMK26-1A-Luciferase construct. The accuracy of each construct was confirmed by DNA sequencing.

Cloning and expression of GFP constructs in HeLa cells. 1A-, 1B-, 1B(Δ16-31)-, and 1A(ins16-31)-UTRs were amplified using primers carrying NheI and BamHI restriction site, respectively at the 5' end and 3' end. The PCR products were cloned upstream of EGFP gene in pEGFP-N1 vector. 5'UTR-GFP constructs were used for transfection of HeLa cells. The accuracy of constructs was confirmed by DNA sequencing.

Yeast strains and growth conditions

The *S. cerevisiae* M3 strain (*MAT*a *lys2 his4 trp1 ade2 leu2 ura3*) is the parental strain of *Δpor1* strain (*MAT*a *lys2 his4 trp1 ade2 leu2 ura3*, *por1*::Leu2) (a kind gift of M. Forte, Oregon[41]). Yeast cells were grown in YP medium (1% yeast extract and 2% bactopeptone) supplemented with different carbon sources: 2% glucose (YPD) or 3% glycerol (YPG). Synthetic minimal medium (SM) (0,67% yeast nitrogen base without amino acids) was supplemented with 2% glucose (SD) or 3% glycerol (SG) and different nutrients, as required. In the drop serial dilution assay, yeast cells were grown on appropriate medium until they reached the exponential phase. The number of cells was determined by optical density at 600nm (1 OD_{600} = 2 x 10^7 cell/ml). Serial dilutions of original culture were applied to Petri dishes. They were next incubated at the indicated temperature for 3-6 days.

Yeast lysates preparation

Yeast cells were grown in 50 ml of YPD medium for 12 hours at 30 °C, under 200 rpm shaking. The cells were harvested by centrifugation for 5 minutes at 3000 x *g*, at 25 °C and then washed twice with sterilized water. The cellular pellet was resuspended in 400 μl of lysis buffer (100 mM Tris–HCl, pH 7.5, 5 mM EDTA, 150 mM NaCl) and lysed in the presence of 1 volume of glass beads 425–600 μm (Sigma) by vortexing in ice 10 times for 60 seconds with 60 seconds intervals. Glass beads were eliminated by centrifugation at 4.500 rpm for 5 minutes and the supernatant was centrifuged 20 minutes at 12000 rpm at 4 °C to obtain the final lysate.

Production of antibodies and immunoblotting

Anti-*D. melanogaster* mt-porin polyclonal antibodies were generated in rabbits by using purified *D. melanogaster* recombinant porin1/VDAC1 as the antigen[2]. Rabbits were were immunized three times, following standard protocols. 100 μg of purified porin were used in any booster. After three immunization cycles, blood was collected and the serum was obtained. Yeast protein extracts were resolved on an SDS-PAGE and electroblotted onto PVDF. Proteins were detected by enhanced chemiluminescence (ECL; Amersham Life Science). Incubation with the anti-*D.m.*mtPorin polyclonal antibody (1:500) was overnight at 4 °C. The secondary antibody (anti-rabbit

immunoglobulin horseradish peroxidase conjugate; Promega) was used 1:5000. Quantification of the protein signals was performed by scanning the immunoblot films using Fluor-S™ MultiImager and Quantity One-4.2.1 software (BioRad). Mitochondrial control was a mouse anti-HSP60 antibody (1:1000) (Abcam), cytosolic control a mouse anti-PGK (1:500) (Novex). Recombinant, or fly-purified (ref. 36 in the text) , *D.m.* VDAC protein was used as positive control.

Fluorescence spectrophotometer and confocal microscopy

Alternative 5' UTRs were cloned in frame at the C-terminal end with the green fluorescent protein gene in pYX212 (Ingenius) and expressed in M22.2 yeast strain[3]. Mid-log-phase cells were analysed using Carl Zeiss 510 UV confocal laser scanning microscope. Fluorescence emission was quantified by using a fluorescence spectrophotometer (Hitachi F2000).

Flow cytometry

20,000 cells per sample were analysed using a CyFlow® ML flow cytometer (Partec) system equipped with three laser sources and 10 optical parameters with dedicated filter setting and a high numerical aperture microscope objective (50x NA 0.82) for the detection of different scatter and fluorescence signals. The cells were excited by an air-cooled argon 488 nm laser and the signal from GFP was read on FL1 detector in log mode. Data obtained were acquired and gated using the FlowMax software (Partec). Fluorescence curves were analysed and plotted by using the FCS Express 5 software (DeNovo). Data are presented as histograms showing the percentage of GFP positive cells, based on the M1 region, calculated by using the FCS Express 5 Flow Research edition. The M1 region was chosen considering the fluorescence intensity shift between untrasfected cells and GFP transfected cells. Values from the flow cytometry are expressed as mean±SEM, representatives for three sets of independent experiments performed in triplicate and based on 20,000 (cells for each sample). Data were statistically analysed by one-way ANOVA.

Mass spectrometry analysis and protein identification

Protein samples from RNA pull-down experiments were then resolved in 12% SOD-PAGE. After Blue Comassie staining, each protein band in each lane was excised from gel and cut in very thin slices. Proteins in gel slices were identified as in[4].

Supplementary Tables

Primers for qRT-PCR	FW5'UTR1A	5'-GTTTGTCGTGGTGTCTCTGTT-3'
	REV-DM-PORIN-167	5’-TCTTCAGATCGAGCTTCCACA-3’
	FW5'UTR1B	5'-TTCGTTATCGTCATGTTGCCA-3'
	REV-DM-PORIN-43	5’-TGTTTGCCCAAATCGCTGTAT-3’
	FW-DM-ACTIN	5’-ACGAGTTGCCCGATGGACAG-3’
	REV-DM-ACTIN	5’-GCACAGTGTTGGCGTACAGA-3’
	FW-PORIN-221	5’-GGAACACAGACAACACGCTG-3’
	REV-PORIN-377	5’-GAATCGGCCTTGACGTTCTC-3’
	FW-ACT1	5’-GCCTTCTACGTTTCCATCCA-3’
	REV-ACT1	5’-GGCCAAATCGATTCTCAAAA-3’
Primers for cloning in pYX212 vector	FW-5’UTR1A-ECORI	5’-TTTTGAATTCACAGAATTACAAAATTTTTTGGTGT-3’
	FW-5’UTR1B-ECORI	5’-TTTTGAATTCTTCCGTTCGTTATCG-3’
	REV-DMPORIN-1A-HINDIII	5’-TTTTAAGCTTCGTTTAATAGGTAAATGTATTTTTAC-3’
	REV-DMPORIN-1B-HINDIII	5’-TTTTAAGCTTGTTTTTAAAGAATATCGTATCTTTT-3’
	REV-ECOR1-1B	5’-AAAGAATTCCTCCCCACATGACC -3’
	REV-ECOR1-1A	5’-AAAGAATTCTTTGATGAGTTTTAGTTTGGTTAGAGT-3’
	FW-GFP	5’-AAAAAGAATTCATGAGTAAAGGAGAAGAA-3’
	REV-GFP	5’-AAAAACTCGAGTTATTTGTATAGTTCATCCAT-3’
	FW-YMC2	5’-ATGAGTGAAGAATTTCCTA-3’
	REV-YMC2	5’-CTACTCTTCCCCCAGAAATC-3’
	DM VDAC-BAMHI-FW	5’-CGCGGATCCATGCTCCTCCATCATACAG-3’
	DM VDAC-HINDIII-	5’-CGCAAGCTTTTATTAGGCCTCCAGCTCCAG-3’
Primers for cloning in pMK26-Luciferase vector	FW-Luciferase	5’-AAAAAAAAGCTTATGGAAGACGCCAAAAACAT-3’
	REV-Luciferase	5’-AAAAAACTCGAGTTACACGGCGATCTTTCC-3’
	FW-1Ains16-31	5’- GTGTACGTTTGTCGTTCATGTTGCCATCCACGGTGTCTCTGTTCCTC-3’
	REV-1A-ins16-31	5’-GAGGAACAGAGACACCGTGGATGGCAACATGAACGACAAACGTACAC 3’
	FW-5’UTR1A-ECORV	5’-TTTTGATATCACAGAATTACAAAATTTTTTGGTGT-3’
	FW-5’UTR1B-ECORV	5’-TTTTGATATCTTCCGTTCGTTATCG-3’
	REV-5’UTR1A-HINDIII	5’-TTTTAAGCTTTTTGATGAGTTTTAGTTTGGTTAGAGT-3’
	REV-5’UTR1B-HINDIII	5’-TTTTAAGCTTCTCCCCACATGACCACAAAC-3’
Primers for mutagenesis	FW-1BΔ1-15	5’-GGCTGCAGGAATTCTAGTGATTTCATGTTGCCATCCACCAGCG-3’
	REV-1BΔ1-15	5’-CGCTGGTGGATGGCAACATGAAATCACTAGAATTCCTGCAGCC-3’
	FW-1BΔ16-31	5’-GTGATTTTCCGTTCGTTATCGCAGCGTGCAACTCGCTCAATA-3’
	REV-1BΔ16-31	5’-TATTGAGCGAGTTGCACGCTGCGATAACGAACGGAAAATCAC-3’
	FW-1BΔ32-47	5’-TCGTCATGTTGCCATCCACCAATAAACATTTTCGATTTC-3’
	REV-1BΔ32-47	5’-GAAATCGAAAATGTTTATTGGTGGATGGCAACATGACGA-3’
	FW-1BΔ48-62	5’-CATCCACCAGCGTGCAACTCGCTATTTCACTCTTTACCGTTCTTT-3’
	REV-1BΔ48-62	5’-AAAGAACGGTAAAGAGTGAAATAGCGAGTTGCACGCTGGTGGATG-3’
	FW-1BΔ63-80	5’-GCTCGCTCAATAAACATTTTCGCTTTATCGTCGTTTTGTTTG-3’
	REV-1BΔ63-80	5’-CAAACAAAACGACGATAAAGCGAAAATGTTTATTGAGCGAGC-3’
	FW-1BΔ79-90	5’-TTTCGATTTCACTCTTTACCGGTTTTGTTTGTTTGTGGTCATGTG-3’
	REV-1BΔ79-90	5’-CACATGACCACAAACAAACAAAACCGGTAAAGAGTGAAATCGAAA-3’
	FW-1BΔ91-105	5’-ACTCTTCACCGTTCTTTATCGTCGGTCATGTGGGGAGATGGCTCC-3’
	REV-1BΔ91-105	5’-GGAGCCATCTCCCCACATGACCGACGATAAAGAACGGTGAAGAGT-3’
	FW-1BΔ106-119	5’-TTATCGTCGTTTTGTTTGTTTGTATGGCTCCTCCATCATACAGCG-3’
	REV-1BΔ106-119	5’-CGCTGTATGATGGAGGAGCCATACAAACAAACAAAACGACGATAA-3’
	FW-1BMUT-ATG	5’-CGTTCGTTATCGTCACGTTGCCATCCACCA-3’

	REV-1BMUT-ATG	5’-TGGTGGATGGCAACGTGACGATAACGAACG-3’
	FW-1BMUT-TAA	5’-CGTGCAACTCGCTCAAGCAACATTTTCGATTTCAC-3’
	REV-1BMUT-TAA	5’-GTGAAATCGAAAATGTTGCTTGAGCGAGTTGCACG-3’
	FW-1BΔ16-28	5'-GTGATTTTCCGTTCGTTATCGCACCAGCGTGCAACTCGCTC-3'
	REV-1BΔ16-28	5'-GAGCGAGTTGCACGCTGGTGCGATAACGAACGGAAAATCAC-3'
	FW-1BΔ19-31	5'-GATTTTCCGTTCGTTATCGTCACAGCGTGCAACTCGCTCAATA-3'
	REV-1BΔ19-31	5'-TATTGAGCGAGTTGCACGCTGTGACGATAACGAACGGAAAATC-3'
	FW-1BΔ19-28	5'-TTCCGTTCGTTATCGTCACACCAGCGTGCAACTCGC-3'
	REV-1BΔ19-28	5'-GCGAGTTGCACGCTGGTGTGACGATAACGAACGGAA-3'
Primers for cloning in pEGFP-N1 vector	5'UTR1A-NHE1-FW	5'-AAAGCTAGCACAGAATTACAAAATTTTTTGGTGT-3’
	5'UTR1A-BAMH1-REV	5'-AAAGGATCCTTTGATGAGTTTTAGTTTGGTTAGA-3'
	5'UTR1B-NHE1-FW	5'-AAAGCTAGCTTCCGTTCGTTATCGTCATGT-3'
	5'UTR-1Bdelta16-31-NHE1-FW	5'-AAAGCTAGCTTCCGTTCGTTATCGCAGCG-3'
	5'UTR1B-BAMH1-REV	5'-AAAGGATCCCTCCCCACATGACCACAAAC-3'

Table S1. List of the primer sequences used in this work.

1B-5'UTR			1A-5'UTR		
**RBP**	**score**	**position (nt)**	**RBP**	**score**	**position (nt)**
**ARET**	high	98-106	**CG17838**	med./high	30-32
**BRU-3**	medium	96-112	**CNOT4**	medium	29
**CG5213**	low	83	**ELAV**	medium	34-39
**CG7903**	medium	106	**ARET**	med./high	39-73
**ELAV**	HIGH	83-102	**BRU-3**	med./high	39-55
**FNE**	varable	varie	**FNE**	medium	37-38
**LARK**	medium	46	**HOW**	high	90
**ORB2**	high	96-105	**PAPI**	med./high	47-61
**PAPI**	high	98-106	**ROX8**	med./low	32-34
**RBP1-like**	med./high	35	**RBP9**	medium	37-38
**RBP9**	variable	varie	**SF1**	high	90
**ROX8**	medium	59-66	**SM**	low	86
**SXL**	med./high	83.103	**SXL**	medium	33-36
**U2AF50**	medium	84-103	**U2AF50**	medium	34-36

Table S2. RNA-Binding Proteins identified by Bionformatic screening on the *D. melanogaster* database at RBPMap server (http://rbpmap.technion.ac.il/). Proteins with high affinity to 1A- or 1B-5’ UTR sequences were identified as RNA-Binding Proteins (RBPs). Many putative RBPs are common between 1A- and 1B-UTRs and only few RBPs are specific.

List of proteins interacting with 10-37 sequence from 1B-VDAC mRNA identified by MS analysis							
**MW**	**PI**	**Protein name/Symbol**	**Score**	**Cover %**	**UniProt ID**	**GO biological process**	**Loc.**
42,3	7,68	K7 Arc1p	707	45	G2WDZ5	tRNA binding	Cyt
96,7	5,66	Valyl-tRNA synthetase (Vas1p)	627	14	E7NHX9	tRNA ligase activity	Cyt
48,72	5,89	Met17p	473	24	E7Q715	Methionine biosynthesis	Cyt
		Hsp70 ATPase SSB2 (Hsp76)	415	19	P40150	Regulation of translational fidelity	Cyt
83,0	8,30	Gus1p	413	17	J8Q5E4	Nucleotide binding	Cyt
44,9	6,13	Oye2p	292	14	E7KDJ6	Oxidoreductase activity	Cyt
44,48	5,02	eIF4a	690	27	P10081	Regulation of translation initiation / unwind RNA secondary structure	Cyt
34,7	5,64	Asc1	203	22	P38011	Negative regulation of translation / bound to 40S ribosome near to exit channel for mRNA	Cyt
25,12	6,52	Gsp2P/Ran-GTPase	659	30	ETM10	Transport of mRNA from nucleus to cytosol	Nc
14,67	9,94	S22A	245	32	A6ZQE6	Ribosomal 40S subunit protein	Cyt
11,68	5,43	Hsp12	255	43	E7KM26	Heat shock protein	Cyt
18,47	5,4	HYP2 (eIF5a)	483	28	P23301	Regulation of translation initiation	Cyt

Table S3. List of proteins interacting with high affinity to 1B 10-37 RNA oligo identified by Mass Spectrometry analysis. Proteins interacting with the 1B 74-95 RNA oligo were also identified by MS analysis with the aim to identify unspecific RBPs able to recognize any RNA molecule. These latter proteins were then subtracted from the whole list of proteins interacting with the 10-37 RNA oligo.

Supplementary Figures

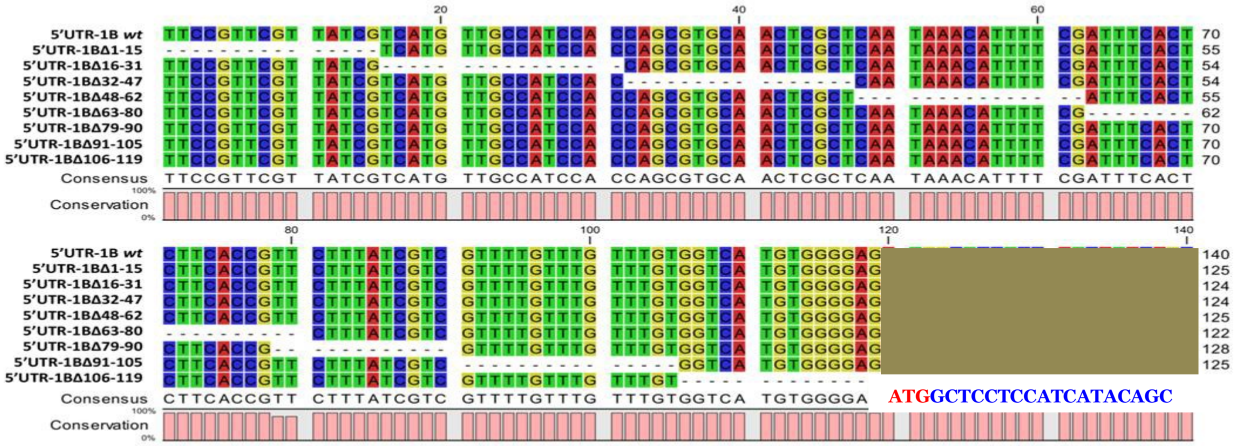

Figure S1. Multiple alignment of 1B-5'UTR wild-type and 1B-5'UTR mutants of *D. melanogaster* porin1. Sequential deletion of 15 nucleotides stretches from 1B-5' UTR region of *D. melanogaster porin1* gene, generated eight different mutant sequences. The multi-alignment reported show the deletions in the 5' UTR for each 1B-VDAC mutant obtained.

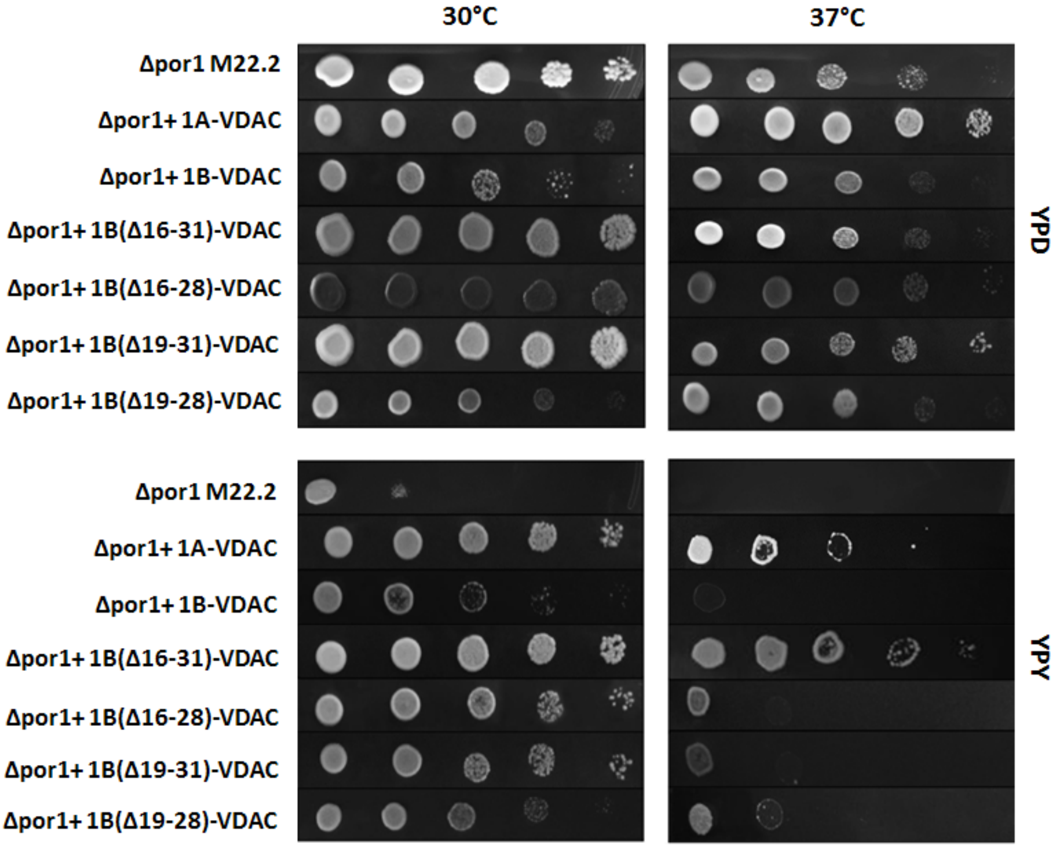

Figure S2. Microtiter assay of Δ*por1 S. cerevisiae* strain transformed with 1A-VDAC, 1B-VDAC or with mutants of 1B-5' UTR region of *D. melanogaster* VDAC gene. Representative panel of Drop-serial dilutions assay of *Δpor1* yeast transformed with 1B-VDAC, 1B(Δ16-31)-VDAC or with mutants generated by deletion of nucleotide surrounding the sequence 16-31 of 1B-5'UTR region: 1B(Δ19-31); 1B(Δ16-28); 1B(Δ19-28). Yeast samples were plated on YPD (2% glucose) or YPY (3% glycerol) and incubated at 30 °C or 37 °C. No major differences were identified in yeast cells growth on YPD at both temperatures. The growth defect of Δ*por1* on YPY at both temperature was fully restored only in cells transformed with 1B(Δ16-31)-VDAC.

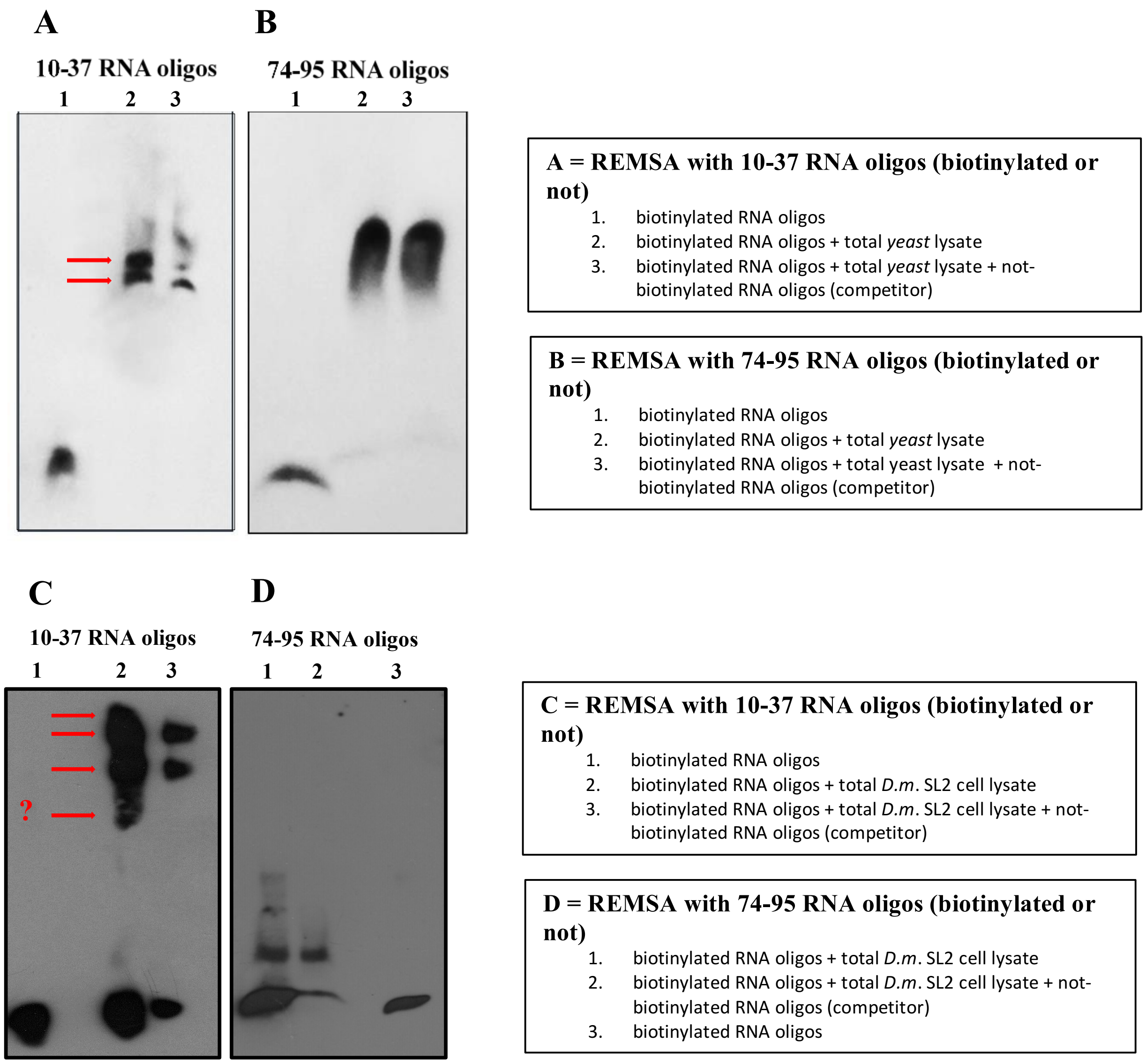

Figure S3. Interaction between RNA oligos derived from specific 1B-5' UTR regions and proteins from yeast or *D.m.* total extracts.

REMSA assay was performed using 10-37 RNA and 74-95 RNA (corresponding to 10-37 and 74-95 sequences of 1B-5' UTR, respectively) as oligos probes against total yeast (A-B) or *D.melanogaster* (C-D) extracts. Following incubation of proteins with RNA, samples were electrophoresed, transferred on a nylon membrane and biotinylated RNA detected by HRP-conjugated streptavidin and autoradiography. In panels A and C, the samples reported are: 1) 0.5 nM of naked biotin-labelled 10-37 RNA; 2) 0.5 nM of biotin-labelled 10-37 RNA and 4 µg of wt yeast (A) or *D. melanogaster.* (C) lysate; 3) 0.5 nM of biotin-labelled 10-37 RNA and 4 µg of wt yeast (A) or *D.m.* (C) lysate and 6 µM of 10-37 RNA competitor. In panels B and D samples are: 4) 2 nM of naked biotin-labelled 74-95 RNA; 5) 2 nM of biotin-labelled 74-95 RNA and 4 µg of wt yeast (B) or *D .melanogaster* (D) lysate; 6) 2 nM of biotin-labelled 74-95 RNA and 4 µg of wt yeast (B) or *D.m.* (D) lysate and 6 µM of 74-95 RNA competitor. Strong and specific interactions were revealed between the 10-37 RNA, encompassing the corresponding 1B(16-31)-5' UTR region, and yeast or *D. melanogaster* extracts A and C, respectively): specific proteins bind to 10-37 oligo and shift the position of RNA band, in comparison to RNA band in lanes 1. The signal shift obtained with 10-37 oligo results from specific protein:RNA interaction because it can be prevented by competition with an excess of unlabeled RNA. No specific protein binding was obtained with the control 74-95 RNA (B,D).

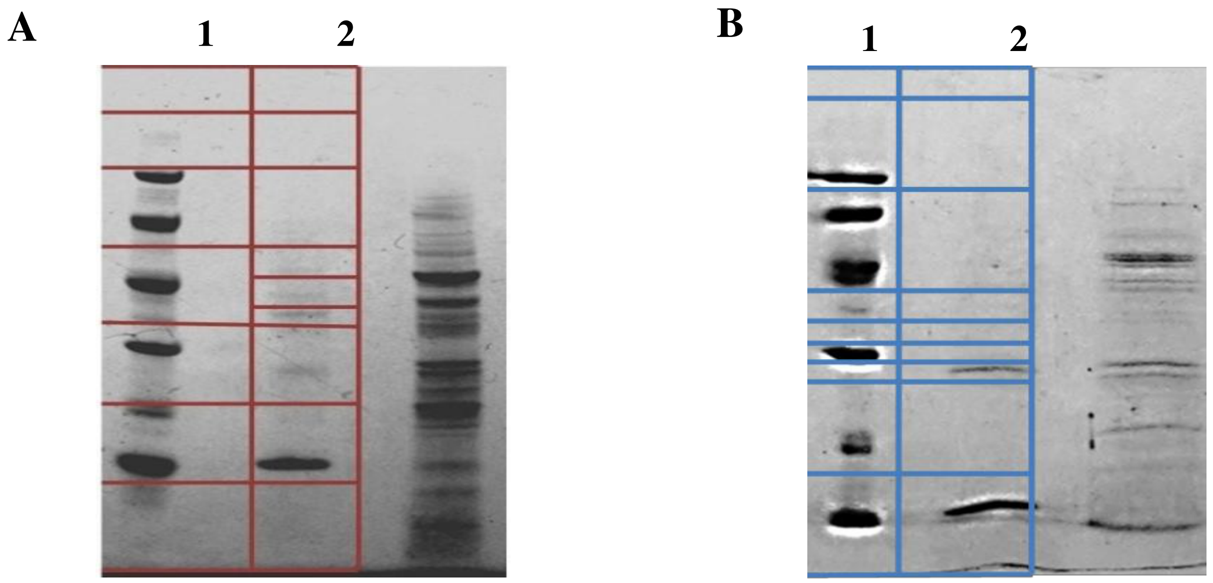

Figure S4. SDS-PAGE analysis of yeast proteins interacting with biotinylated 10-37 RNA oligo. Panel A: 1) protein ladder; 2) proteins interacting with the biotinylated 10-37 RNA oligo; 3) proteins not interacting with the biotinylated 10-37 RNA oligo. Panel B: 1) protein ladder; 2) proteins interacting with the biotinylated 74-95 RNA oligo; 3) Proteins not interacting with the biotinylated 74-95 RNA oligo.

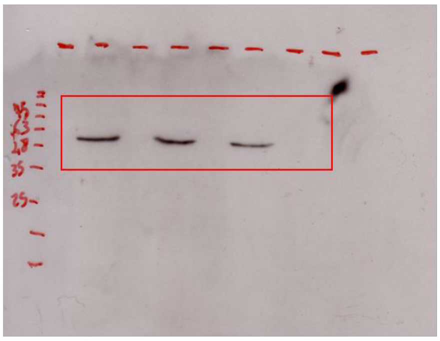

HSP60

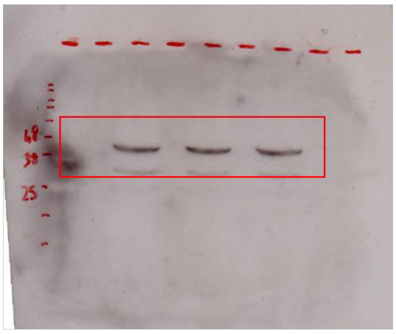

PGK

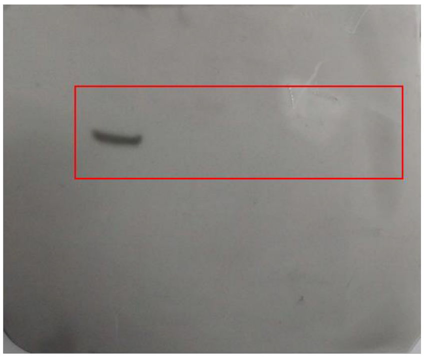

1A-VDAC

Fig. S5. Full scan of western blot shown in Fig. 2F.

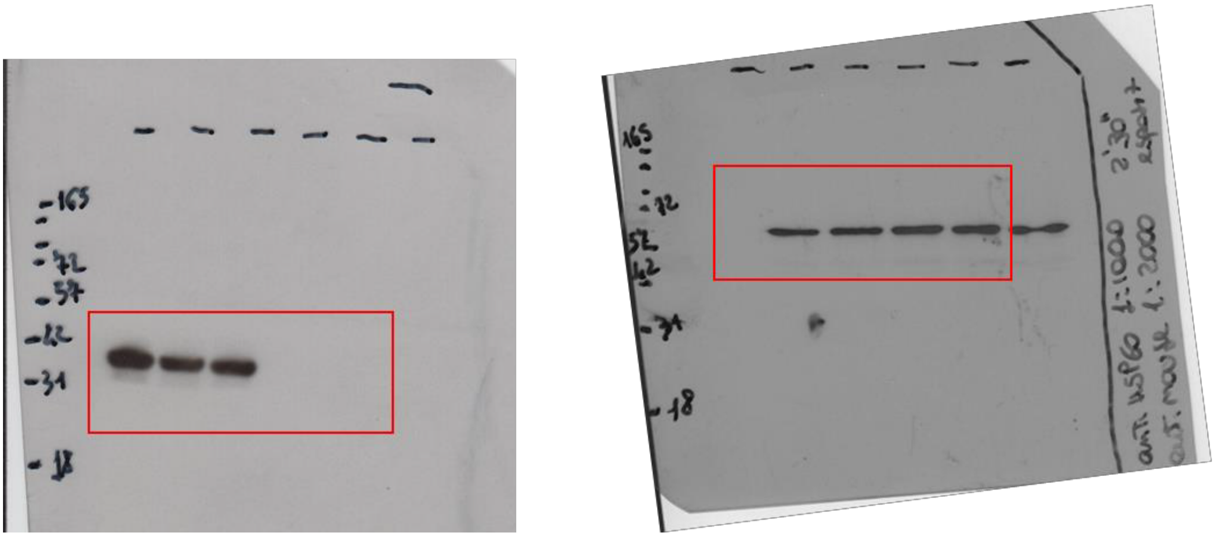

Fig. S6. Full scan of western blot shown in Fig. 3E.

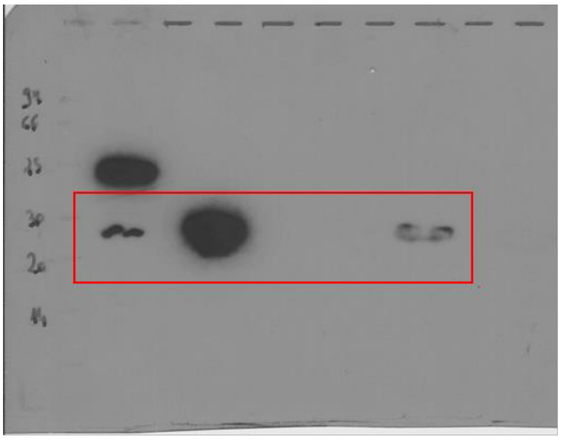

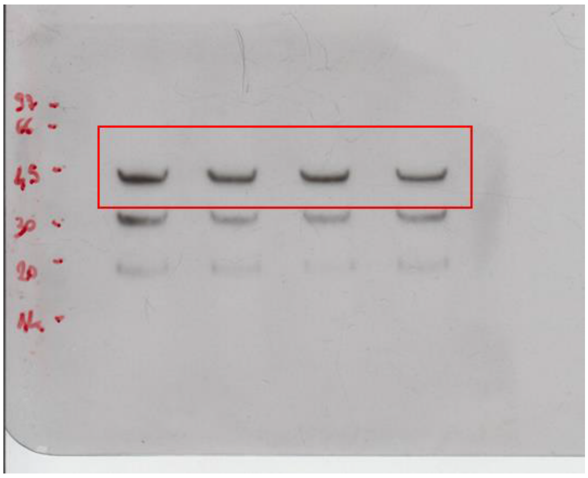

Fig. S7. Full scan of western blot shown in Fig. 3F

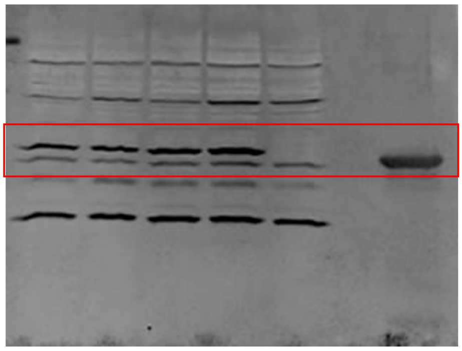

1A-VDAC

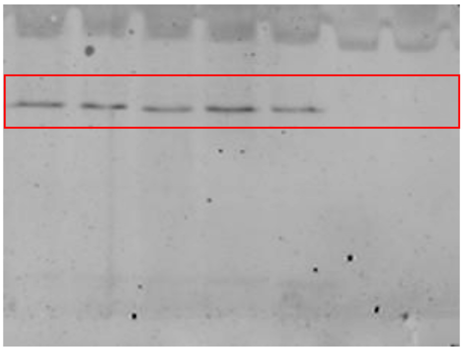

TUBULIN

Fig. S8. Full scan of western blot shown in Fig. 5D.

Supplementary References

1. Checchetto, V., Reina, S., Magrì, A., Szabo, I. & De Pinto, V. Recombinant human voltage dependent anion selective channel isoform 3 (hVDAC3) forms pores with a very small conductance. *Cell Physiol Biochem* **34,** 842-853 (2014).

2. Guarino, F. *et al*. Expression and localization in spermatozoa of the mitochondrial porin isoform 2 in *Drosophila melanogaster*. *Biochem Biophys Res Commun* **346,** 665–670 (2006).

3. Newman S.M., Zelenaya T.O., Perlman P.S. & Butow R.A. Analysis of mitochondrial DNA nucleoids in wild-type and a mutant strain of *Saccharomyces cerevisiae* that lacks the mitochondrial HMG box protein Abf2p. *Nucleic Acids Res* **24**, 386-393 (1996).

4. Messina, A. *et al*. Live cell interactome of the human voltage dependent anion channel 3 (VDAC3) revealed in HeLa cells by affinity purification tag technique. *Mol Biosyst.* **8,** 2134-45 (2014).
